# Supplementary material for: Structural elucidation of recombinant Trichomonas vaginalis 20S proteasome bound to covalent inhibitors
Source: Nat Commun. 2024 Oct 4;15:8621. doi: 10.1038/s41467-024-53022-w (PMC11452676; doi:10.1038/s41467-024-53022-w)
Supplement: Supplementary file 1 — Supplementary Information [file 41467_2024_53022_MOESM1_ESM.pdf]

Supplementary Information:

Structural elucidation of recombinant *Trichomonas vaginalis* 20S proteasome bound to covalent inhibitors

Jan Silhan[1,#], Pavla Fajtova[1,2,#,*], Jitka Bartosova[1], Brianna M. Hurysz[2], Jehad Almaliti[3], Yukiko Miyamoto[4], Lars Eckmann[4], William Gerwick[3], Anthony J. O'Donoghue[2,5*], Evzen Boura[1,*]

[1]Institute of Organic Chemistry and Biochemistry AS CR, v.v.i., Flemingovo nam. 2., 166 10 Prague 6, Czech Republic
[2]Skaggs School of Pharmacy and Pharmaceutical Sciences, University of California San Diego, La Jolla, CA, 92037, USA
[3]Scripps Institution of Oceanography, University of California San Diego, La Jolla, CA, 92037, USA
[4]Division of Gastroenterology, School of Medicine, University of California San Diego, La Jolla, CA, 92037, USA
[5]Center for Discovery and Innovation in Parasitic Diseases, Skaggs School of Pharmacy and pharmaceutical Sciences, University of California, San Diego, 9500 Gilman Dr., La Jolla, CA 92093, USA

[#]these authors contributed equally
*correspondence to: fajtova@uochb.cas.cz or ajodonoghue@health.ucsd.edu or boura@uochb.cas.cz

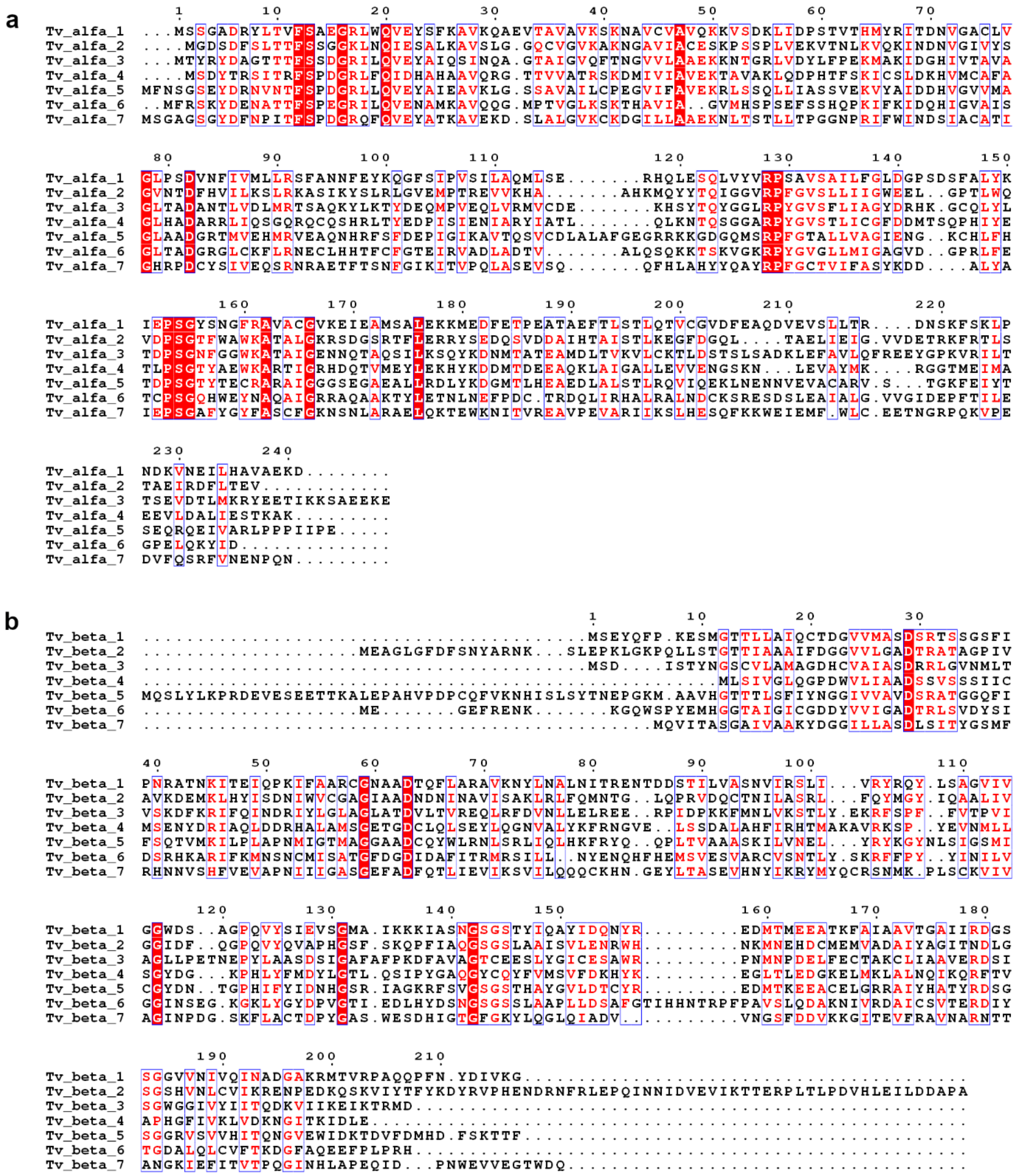
a
Tv_alfa_1
Tv_alfa_2
Tv_alfa_3
Tv_alfa_4
Tv_alfa_5
Tv_alfa_6
Tv_alfa_7
b
Tv_beta_1
Tv_beta_2
Tv_beta_3
Tv_beta_4
Tv_beta_5
Tv_beta_6
Tv_beta_7

c

```
              1        10        20        30        40        50        60        70
Tv_alfa_1  ....MSSGADRYLTVFSAEGRLWQVEYSFKAVKQAEVTAVAVKSKNAVCVAVQKKVSDKLIDPSTVTHMYRITDNVGACL
Hs_alfa_1  MSRGSSAGFDRHITIFSPEGRLYQVEYAFKAINQGGLTSVAVRGKDCAVIVTQKKVPDKLLDSSTVTHLFKITENIGCVM

             80        90       100       110       120       130       140       150
Tv_alfa_1  VGLPSDVNFIVMLLRSFANNFEYKQGFSIPVSILAQMLSERHQLESQLVYVRPSAVSAILFGLDGPSDSFALYKIEPSGY
Hs_alfa_1  TGMTADSRSQVQRARYEAANWKYKYGYEIPVDMLCKRIADISQVYTQNAEMRPLGCCMILIGIDEEQ.GPQVYKCDPAGY

            160       170       180       190       200       210       220       230
Tv_alfa_1  SNGFRAVACGVKEIEAMSALEKKMEDFE..TPEATAEFTLSTLQTVCGVDFEAQDVEVSLLTRDNSKFSKLPNDKVNEIL
Hs_alfa_1  YCGFKATAAGVKQTESTSFLEKKVKKKFDWTFEQTVETAITCLSTVLSIDFKPSEIEVGVVTVENPKFRILTEAEIDAHL

             240
Tv_alfa_1  HAVAEKD
Hs_alfa_1  VALAERD


             1        10        20        30        40        50        60        70
Tv_alfa_2  ..MGDSDFSLTTFSSGGKLNQIESALKAVSLGGQCVGVKAKNGAVIACESKPSSPLVEKVTNLKVQKINDNVGIVYSGVN
Hs_alfa_2  MAERGYSFSLTTFSPSGKLVQIEYALAAVAGGAPSVGIKAANGVVLATEKKQKSILYDERSVHKVEPITKHIGLVYSGMG

             80        90       100       110       120       130       140       150
Tv_alfa_2  TDFHVILKSLRKASIKYSLRLGVEMPTREVVKHAAHKMQYYTQIGGVRPFGVSLLIIGWEELGPTLWQVDPSGTFWAWKA
Hs_alfa_2  PDYRVLVHRARKLAQQYYLVYQEPIPTAQLVQRVASVMQEYTQSGGVRPFGVSLLICGWNEGRPYLFQSDPSGAYFAWKA

           160       170       180       190       200       210       220       230
Tv_alfa_2  TALGKRSDGSRTFLERRYSEDQSVDDAIHTAISTLKEGFDGQLTAELIEIGVVDETRKFRTLSTAEIRDFLTEV.
Hs_alfa_2  TAMGKNYVNGKTFLEKRYNEDLELEDAIHTAILTLKESFEGQMTEDNIEVGICNEA.GFRRLTPTEVKDYLAAIA


           1        10        20        30        40        50        60        70
Tv_alfa_3  MTYRYDAGTTTFSSDGRILQVEYAIQSINQAGTAIGVQFTNGVVLAAEKKNTGRLVDY.LFPEKMAKIDGHIVTAVAGLT
Hs_alfa_3  MSRRYDSRTTIFSPEGRLYQVEYAMEAIGHAGTCLGILANDGVLLAAERRNIHKLLDEVFFSEKIYKLNEDMACSVAGIT

           80        90       100       110       120       130       140       150
Tv_alfa_3  ADANTLVDLMRTSAQKYLKTYDEQMPVEQLVRMVCDEKHSYTQYGGLRPYGVSFLIAGYDRHKGCQLYLTDPSGNFGGWK
Hs_alfa_3  SDANVLTNELRLIAQRYLLQYQEPIPCEQLVTALCDIKQAYTQFGGKRPFGVSLLYIGWDKHYGFQLYQSDPSGNYGGWK

          160       170       180       190       200       210       220       230
Tv_alfa_3  ATAIGENNQTAQSILKSQYKDN.MTATEAMDLTVKVLCKTLDSTSLSADKLEFAVLQFREEYGPKVRILTTSEVDTLMKR
Hs_alfa_3  ATCIGNNSAAAVSMLKQDYKEGEMTLKSALALAIKVLNKTMDVSKLSAEKVEIATLTR.ENGKTVIRVLKQKEVEQLIKK

          240       250
Tv_alfa_3  YEETIKKSAEEKE..........
Hs_alfa_3  HEEEEAKAEREKKEKEQKEKDK


           1        10        20        30        40        50        60        70        80
Tv_alfa_4  MSDYTRSITRFSPDGRLFQIDHAHAAVQRGTTVVATRSKDMIVIAVEKTAVAKLQDPHTFSKICSLDKHVMCAFAGLHAD
Hs_alfa_4  .MSYDRAITVFSPDGHLFQVEYAQEAVKKGSTAVGVRGRDIVVLGVEKKSVAKLQDERTVRKICALDDNVCMAFAGLTAD

                       90       100       110       120       130       140       150       160
Tv_alfa_4  ARLIQSGQRQCQSHRLTYEDPISIENIARYIATLQLKNTQSGGARPYGVSTLICGFDDMTSQPHIYETLPSGTYAEWKA
Hs_alfa_4  ARIVINRARVECQSHRLTVEDPVTVEYITRYIASLKQRYTQSNGRRPFGISALIVGFD.FDGTPRLYQTDPSGTYHAWKA

                     170       180       190       200       210       220       230
Tv_alfa_4  RTIGRHDQTVMEYLEKHYKDDMTD..EEAQKLAIGALLEVVENGSKNLEVAYMKRGGTMEIMAEEVLDALIESTKAK...
Hs_alfa_4  NAIGRGAKSVREFLEKNYTDEAIETDDLTIKLVIKALLEVVQSGGKNIELAVMRRDQSLKILNPEEIEKYVAEIEKEKEE

Tv_alfa_4  ..........
Hs_alfa_4  NEKKKQKKAS


           1        10        20        30        40        50        60        70        80
Tv_alfa_5  MFNSGSEYDRNVNTFSPDGRLLQVEYAIEAVKLGSSAVAILCPEGVIFAVEKRLSSQLLIASSVEKVYAIDDHVGVVMAG
Hs_alfa_5  MFLTRSEYDRGVNTFSPEGRLFQVEYAIEAIKLGSTAIGIQTSEGVCLAVEKRITSPLMEPSSIEKIVEIDAHIGCAMSG

                       90       100       110       120       130       140       150       160
Tv_alfa_5  LAADGRTMVEHMRVEAQNHRFSFDEPIGIKAVTQSVCDLALAFGEGRRKKGDGQMSRPFGTALLVAGIENGKCHLFHTDP
Hs_alfa_5  LIADAKTLIDKARVETQNHWFTYNETMTVESVTQAVSNLALQFGEED..ADPGAMSRPFGVALLFGGVDEKGPQLFHMDP

                     170       180       190       200       210       220       230       240
Tv_alfa_5  SGTYTECRARAIGGGSEGAEALLRDLYKDGMTLHEAEDLALSTLRQVIQEKLNENNVEVACARVSTGKFEIYTSEQRQEI
Hs_alfa_5  SGTFVQCDARAIGSASEGAQSSLQEVYHKSMTLKEAIKSSLIILKQVMEEKLNATNIELATVQP.GQNFHMFTKEELEEV

                     250
Tv_alfa_5  VARLPPPIIPE
Hs_alfa_5  IKDI.......
```

```
Tv_alfa_6  MFRSKYDENATTFSPEGRILQVENAMKAVQQGMPTVGLKSKTHAVIAGVMHSPSEFSSHQPKIFKIDQHIGVAISGLTAD
Hs_alfa_6  MFRNQYDNDVTVWSPQGRIHQIEYAMEAVKQGSATVGLKSKTHAVLVALKRAQSELAAHQKKILHVDNHIGISIAGLTAD

Tv_alfa_6  GRGLCKFLRNECLHHTFCFGTEIRVADLADTVALQSQKKTSKVGKRPYGVGLLMIGAGVDGPRLFETCPSGQHWEYNAQA
Hs_alfa_6  ARLLCNFMRQECLDSRFVFDRPLPVSRLVSLIGSKTQIPTQRYGRRPYGVGLLIAGYDDMGPHIFQTCPSSANYFDCRAMS

Tv_alfa_6  IGRRAQAAKTYLETNLNEFPDCTRDQLIRHALRALNDCKSRESD.SLEAIALGVVGIDEPFTILEGPELQKYID......
Hs_alfa_6  IGARSQSARTYLERHMSEFMECNLNELVKHGLRALRETLPAEQDLTTKNVSIGIVGKDLEFTIYDDDDVSPFLEGLEERP

Tv_alfa_6  .......................
Hs_alfa_6  QRKAQPAQPADEPAEKADEPMEH
```

```
Tv_alfa_7  MSGAGSGYDFNPITFSPDGRQFQVEYATKAVEKDSLALGVKCKDGILLAAEKNLTSTLLTPGGNPRIFWINDSIACATIG
Hs_alfa_7  MSSIGTGYDLSASTFSPDGRVFQVEYAMKAVENSSTAIGIRCKDGVVFGVEKLVLSKLYEEGSNKRLFNVDRHVGMAVAG

Tv_alfa_7  HRPDCYSIVEQSRNRAETFTSNFGIKITVPQLASEVSQQFHLAHYYQAYRPFGCTVIFASYK...DDALYAIEPSGAFYG
Hs_alfa_7  LLADARSLADIAREEASNFRSNFGYNIPLKHLADRVAMYVHAYTLYSAVRPFGCSFMLGSYSVNDGAQLYMIDPSGVSYG

Tv_alfa_7  YFASCFGKNSNLARAELQKTEWKNITVREAVPEVARIIKSLHES.QFKKWEIEMFWLCEETNGRPQKVPEDVFQSRFV..
Hs_alfa_7  YWGCAIGKARQAAKTEIEKLQMKEMTCRDIVKEVAKIIYIVHDEVKDKAFELELSWVGELTNGRHEIVPKDIREEAEKYA

Tv_alfa_7  NENPQN.........
Hs_alfa_7  KESLKEEDESDDDNM
```

d

```
Tv_beta_1  TTLLAIQCTDGVVMASDSRTSSGSFIPNRATNKITEIQPKIFAARCGNAADTQFLARAVKNYLNALNITRENTDDSTILV
Hs_beta_1  TTIMAVQFDGGVVLGADSRTTTGSYIANRVTDKLTPIHDRIFCCRSGSAADTQAVADAVTYQLGFHSIEL..NEPPLVHT

Tv_beta_1  ASNVIRSLIVRYRQYLSAGVIVGGWDS.AGPQVYSIEVSGMAIKKKIASNGSGSTYIQAYIDQNYREDMTMEEATKFAIA
Hs_beta_1  AASLFKEMCYRYREDLMAGIIIAGWDPQEGGQVYSVPMGGMMVRQSFAIGGSGSSYIYGYVDATYREGMTKEECLQFTAN

Tv_beta_1  AVTGAIIRDGSSGGVVNIVQINADGAKRMTVRPAQQPF.NYDIVKG.
Hs_beta_1  ALALAMERDGSSGGVIRLAAIAESGVERQVLLGDQIPKFAVATLPPA
```

```
Tv_beta_2  TTIAAAIFDGGVVLGADTRATAGPIVAVKDEMKLHYISDNIWVCGAGIAADNDNINAVISAKLRLFQMNTGLQPRVDQCT
Hs_beta_2  TTIAGVVYKDGIVLGADTRATEGMVVADKNCSKIHFISPNIYCCGAGTAADTDMTTQLISSNLELHSLSTGRLPRVVTAN

Tv_beta_2  NILASRLFQYMGYIQAALIVGGIDFQGPQVYQVAPHGSFSKQPFIAQGSGSLAAISVLENRWHNKMNEHDCMEMVADAIY
Hs_beta_2  RMLKQMLFRYQGYIGAALVLGGVDVTGPHLYSIYPHGSTDKLPYVTMGSGSLAAMAVFEDKFRPDMEEEEAKNLVSEAIA

Tv_beta_2  AGITNDLGSGSHVNLCVIKRENPEDKQSKVIYTFYKDYRVPHENDRNF...R........LEPQINNIDVEVIKTTERPL
Hs_beta_2  AGIFNDLGSGSNIDLCVISKNKL.........DFLRPYTVPNKKGTRLGRYRCEKGTTAVLTEKITPLEIEVLEETVQTM

Tv_beta_2  TLPDVHLEILDDAPA
Hs_beta_2  DTS............
```

```
Tv_beta_3  MSDISTYNGSCVLAMAGDHCVAIASDRRLGVNMLTVSKDFKRIFQINDRIYLGLAGLATDVLTVREQLRFDVNLLELREE
Hs_beta_3  .MSIMSYNGGAVMAMKGKNCVAIAADRRFGIQAQMVTTDFQKIFPMGDRLYIGLAGLATDVQTVAQRLKFRLNLYELKEG

Tv_beta_3  RPIDPKKFMNLVKSTLYEKRFSPFFVTPVIAGLLPETNEPYLAASDSIGAFAFPKDFAVAGTGEESLYGICESAWRPNMN
Hs_beta_3  RQIKPYTLMSMVANLLYEKRFGPYYTEPVIAGLDPKTFKPFICSLDLIGCPMVTDDFVVSGTCAEQMYGMCESLWEPNMD

Tv_beta_3  PDELFECTAKCLIAAVERDSISGWGGIVYIITQDKVIIKEIKTRMD
Hs_beta_3  PDHLFETISQAMLNAVDRDAVSGMGVIVHIIEKDKITTRTLKARMD
```

```
Tv_beta_4  MLSIVGLQGPDWVLIAADSSVSSSIICMSENYDRIAQLDDRHALAMSGETGDCLQLSEYLQGNVALYKFRNGVELSSDAL
Hs_beta_4  MEYLIGIQGPDYVLVASDRVAASNIVQMKDDHDKMFKMSEKILLLCVGEAGDTVQFAEYIQKNVQLYKMRNGYELSPTAA

Tv_beta_4  AHFIRHTMAKAVR.KSPYEVNMLLSGYDG..KPHLYFMDYLGTLQSIPYGAQGYCQYFVMSVFDKHYKEGLTLEDGKELM
Hs_beta_4  ANFTRRNLADCLRSRTPYHVNLLLAGYDEHEGPALYYMDYLAALAKAPFAAHGYGAFLTLSILDRYYTPTISRERAVELL

Tv_beta_4  KLALNQIKQRFTVAPHGFIVKLVDKNGITKIDLE.......
Hs_beta_4  RKCLEELQKRFILNLPTFSVRIIDKNGIHDLDNISFPKQGS


Tv_beta_5  TTTLSFIYNGGIVVAVDSRATGGQFIFSQTVMKILPLAPNMIGTMAGGAADCQYWLRNLSRLIQLHKFRYQQPLTVAAAS
Hs_beta_5  TTTLAFKFRHGVIVAADSRATAGAYIASQTVKKVIEINPYLLGTMAGGAADCSFWERLLARQCRIYELRNKERISVAAAS

Tv_beta_5  KILVNELYRYKGYNLSIGSMICGYDNTGPHIFYIDNHGSRIAGKRFSVGSGSTHAYGVLDTCYREDMTKEEACELGRRAI
Hs_beta_5  KLLANMVYQYKGMGLSMGTMICGWDKRGPGLYYVDSEGNRISGATFSVGSGSVYAYGVMDRGYSYDLEVEQAYDLARRAI

Tv_beta_5  YHATYRDSGSGGRVSVVHITQNGVEWIDKTDVFDMHDFSKTTF.
Hs_beta_5  YQATYRDAYSGGAVNLYHVREDGWIRVSSDNVADLHEKYSGSTP


Tv_beta_6  ................ME.GEFRENKKGQWSPYEMHGGTAIGICGDDYVVIGADTRLSVDYSIDSRHKARIFKMNSNCMI
Hs_beta_6  MLSSTAMYSAPGRDLGMEPHRAAGPLQLRFSPYVFNGGTILAIAGEDFAIVASDTRLSEGFSIHTRDSPKCYKLTDKTVI

Tv_beta_6  SATGFDGDIDAFITRMRSILLNYENQHFHEMSVESVARCVSNTLYSKRFFPYYINILVGGINSEGKGKLYGYDPVGTIED
Hs_beta_6  GCSGFHGDCLTLTKIIEARLKMYKHSNNKAMTTGAIAAMLSTILYSRRFFPYYVYNIIGGLDEEGKGAVYSFDPVGSYQR

Tv_beta_6  LHYDSNGSGSSLAAPLLDSAFGTIHHNTRPFPAVSLQDAKNIVRDAICSVTERDIYTGDALQLCVFTKDGFAQEEFPLPR
Hs_beta_6  DSFKAGGSASAMLQPLLDNQVGFKNMQNVEHVPLSLDRAMRLVKDVFISAAERDVYTGDALRICIVTKEGIREETVSLRK

Tv_beta_6  H
Hs_beta_6  D


Tv_beta_7  ..............................................MQVITASGAIVAAKYDGGILLASDLSITYGSMFR
Hs_beta_7  MEAFLGSRSGLWAGGPAPGQFYRIPSTPDSFMDPASALYRGPITRTQNPMVTGTSVLGVKFEGGVVIAADMLGSYGSLAR

Tv_beta_7  HNNVSHFVEVAPNIIIGASGEFADFQTLIEVIKSVILQQQCKHNGEYLTASEVHNYIKRYMYQCRSNMKPLSCKVIVAGI
Hs_beta_7  FRNISRIMRVNNSTMLGASGDYADFQYLKQVLGQMVIDEELLGDGHSYSPRAIHSWLTRAMYSRRSKMNPLWNTMVIGG.

Tv_beta_7  NPDGSKFLACTDPYGASWESDHIGTGFGKYLQGLQIADVVNGSFD....DVKKGITEVFRAVNARNTTANGKIEFITVTP
Hs_beta_7  YADGESFLGYVDMLGVAYEAPSLATGYGAYLAQPLLREVLEKQPVLSQTEARDLVERCMRVLYYRDARSYNRFQIATVTE

Tv_beta_7  QGINHLAPEQIDPNWEVVEGTWDQ.
Hs_beta_7  KGVEIEGPLSTETNWDIAHMISGFE
```

e

```
Tv_Ump-1  .................MYEQWIPEEPVDRLREGLPNLRHGSVNQHPLEIAIEERRKTQFKDKFDELALLYGEGFANHEK
Hs_Ump-1  MNARGLGSELKDSIPVTELSASGPFESHDLLRKGFSCVKNELLPSHPLELSEKNFQLNQDKMNFSTLRNIQGLFAPLKLQ

Tv_Ump-1  MLYKLIKSTRIGFRTYERPDDLAIEVFTGDIDDVDFNDMFAPNGMRADIEFDPHEIQEKRLNIE
Hs_Ump-1  MEFKA..VQQVQRLPFLSSSNLSLDVLRGNDETIGFEDILNDPSQ.SEVMGEPHLMVEYKLGLL
```

f

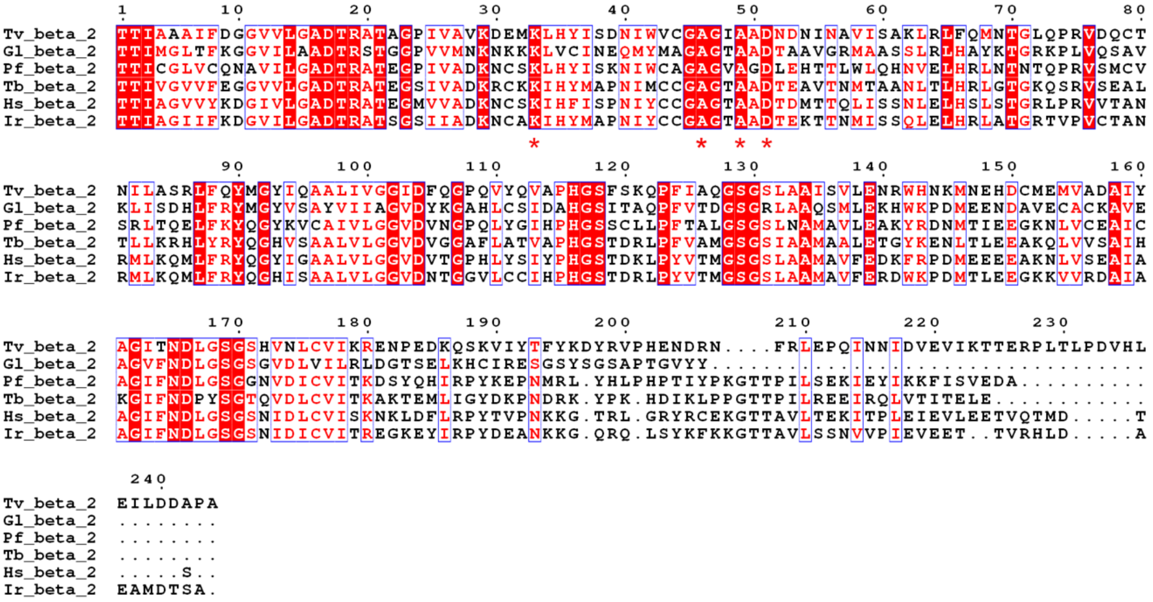

g

Subunit	*Tv* Uniprot ID	*Hs* Uniprot ID
**α1**	A2F568	P60900
**α2**	A2FJV7	P25787
**α3**	A2FT79	P25789
**α4**	A2DTN3	O14818
**α5**	A2FCM7	P28066
**α6**	A2E1I9	P25786
**α7**	A2D8G5	P25788
**β1**	A2E7Z2	P28072
**β2**	A2F2T6	Q99436
**β3**	A2F3H9	P49720
**β4**	A2F8W4	P49721
**β5**	A2DD57	P28074
**β6**	A2F716	P20618
**β7**	A2F3X4	P28070
**Ump-1**	A2FJW0	Q9Y244

Supplementary Figure 1. Sequence alignment of *Tv*20S and human 20S proteasomes and Ump-1 chaperone.

Panel **a)** represents multiple sequence alignments of all seven α subunits (α1-α7) and **b)** all seven β subunits (β1-β7) from *Tv*20S, with β1, β2 and β5 shown as proenzymes, and **c)** & **d)** aligned individually with their human α & β subunits (*Hs*20S) counterparts. Conserved residues are boxed, and identical residues are highlighted in red. Conservation patterns and variations between species are illustrated. **e)** Sequence alignment of *Tv* Ump-1 and with *Hs* Ump-1, highlighting conserved regions. **f)** Sequence alignment of mature β2 subunit from *T. vaginalis (Tv), Giardia lamblia (Gl), Plasmodium falciparum (Pf), Trypanosoma brucei (Tb), Homo sapiens (Hs),* and *Ixodes ricinus (Ir)*. The asterisk denotes highly conserved residues Lys33 and a loop consisting of Ala46, Ala49, and Asn52. The aligned sequences were visualized using ESP3 software according to Robert, X. and Gouet, P. (2014).[1] **g)** Uniprot accession codes of each gene used.

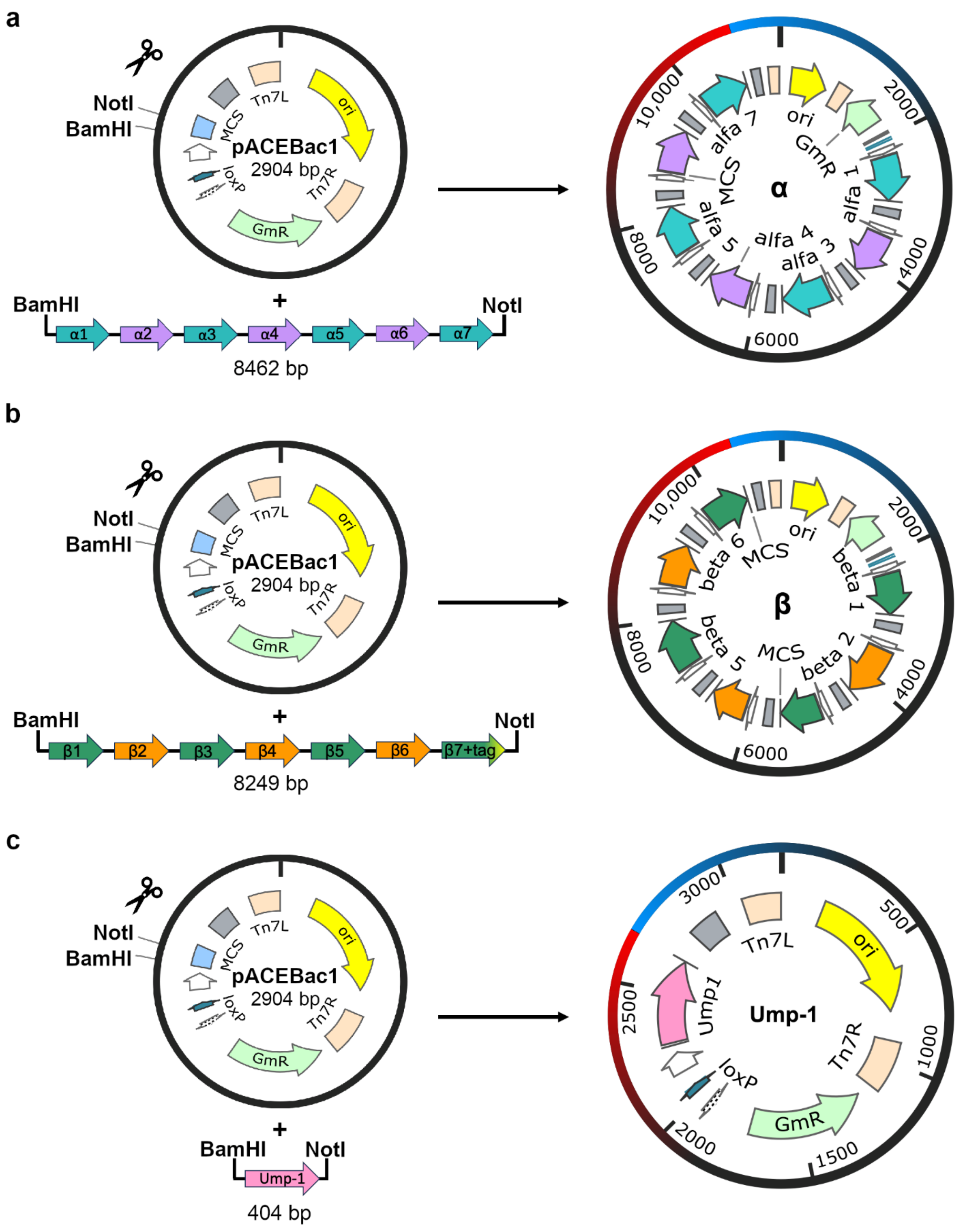
a
NotI
BamHI
pACEBac1
2904 bp
Tn7L
ori
MCS
loxP
Tn7R
GmR
BamHI
+
NotI
α1
α2
α3
α4
α5
α6
α7
8462 bp
α
ori
GmR
alfa 1
alfa 3
alfa 4
alfa 5
MCS
alfa 7
2000
4000
6000
8000
10,000
b
NotI
BamHI
pACEBac1
2904 bp
Tn7L
ori
MCS
loxP
Tn7R
GmR
BamHI
+
NotI
β1
β2
β3
β4
β5
β6
β7+tag
8249 bp
β
ori
beta 1
beta 2
MCS
beta 5
beta 6
MCS
2000
4000
6000
8000
10,000
c
NotI
BamHI
pACEBac1
2904 bp
Tn7L
ori
MCS
loxP
Tn7R
GmR
+
BamHI
NotI
Ump-1
404 bp
Ump-1
Tn7L
ori
Tn7R
GmR
loxP
Ump1
500
1000
1500
2000
2500
3000

***d* Insert BamHI_α1_α2_α3_α4_α5_α6_α7_ NotI** - *sequences encoding Kozak sequence, **start** and **stop** codon, gene, **polyhedrin promoter**, SV40 polyA tail and twin-strep tag.are highlighted.*

*ggatcc*GCCACC**ATG**AGCAGCGGTGCAGATCGTTATCTGACCGTTTTTAGTGCCGAAGGTCGTCTGTGGCAGGTTGAATATAGTTTTAA
AGCAGTTAAACAGGCCGAAGTTACCGCAGTTGCAGTTAAAAGCAAAAATGCAGTTTGTGTTGCCGTGCAGAAAAAGGTTAGCGATAAAC
TGATTGATCCGAGCACCGTTACACACATGTATCGTATTACCGATAATGTTGGTGCATGTCTGGTTGGTCTGCCGAGTGATGTTAACTTT
ATTGTTATGCTGCTGCGTAGCTTTGCCAACAACTTTGAATATAAACAGGGCTTTAGCATCCCGGTTTCAATTCTGGCACAGATGCTGAG
CGAACGTCATCAGCTGGAAAGCCAGCTGGTTTATGTTCGTCCGAGCGCAGTTAGCGCAATTCTGTTTGGTCTGGATGGTCCGAGCGATA
GCTTTGCACTGTATAAAATCGAACCGAGCGGTTATAGCAATGGTTTTCGTGCAGTTGCATGTGGCGTTAAAGAAATTGAAGCAATGAGC
GCACTGGAAAAGAAGATGGAAGATTTTGAAACACCGGAAGCAACCGCAGAATTTACCCTGAGCACCCTGCAAACCGTTTGTGGTGTTGA
TTTTGAAGCACAGGATGTTGAAGTTAGCCTGCTGACCCGTGATAATAGCAAATTTTCAAAACTGCCGAACGACAAGGTGAACGAAATTC
TGCATGCCGTTGCCGAAAAAGAT**TAA**AGATCTAGAGGATCATAATCAGCCATACCACATTTGTAGAGGTTTTACTTGCTTTAAAAAACC
TCCCACACCTCCCCCTGAACCTGAAACATAAAATGAATGCAATTGTTGTTGTTAACTTGTTTATTGCAGCTTATAATGGTTACAAATAA
AGCAATAGCATCACAAATTTCACAAATAAAGCATTTTTTTCACTGCATTCTAGTTGTGGTTTGTCCAAACTCATCAATGTATCTTATCA
TGTCTGGATCTGATCACTGCTTGAGCCTAGAAGATCCGGCTGCTAACAAAGCCCGAAAGGAAGCTGAGTTGGCTGCTGCCACCGCTGAG
CAATAACTATCATAACCCCTAGGGTATACCCATCTAAGGTAGCGAGTTTAAACACTAGTATCGATTCGCGACCTACTCCGGAATATTAA
TAG**ATCATGGAGATAATTAAAATGATAACCATCTCGCAAATAAATAAGTATTTTACTGTTTTCGTAACAGTTTTGTAATAAAAAAACCT**
ATAAATATTCCGGATTATTCATACCGTCCCACCATCGGGCGCGAGCTCGCCACC**ATG**GGCGATAGCGATTTTAGCCTGACCACCTTTAG
CAGCGGTGGTAAACTGAATCAGATTGAAAGCGCACTGAAAGCAGTTAGCTTAGGTGGCCAGTGTGTTGGTGTTAAAGCCAAAAATGGTG
CAGTTATTGCCTGTGAAAGCAAACCGAGCAGTCCGCTGGTTGAAAAAGTTACCAATCTGAAAGTGCAGAAAATCAACGATAATGTGGGC
ATTGTTTATAGCGGTGTGAACACCGATTTTCACGTTATTCTGAAAAGCCTGCGTAAAGCCAGCATCAAATATAGCCTGCGTCTGGGTGT
TGAAATGCCGACACGTGAAGTTGTTAAACATGCAGCACATAAGATGCAGTATTATACCCAGATTGGTGGTGTTCGTCCGTTTGGTGTTA
GCCTGCTGATTATTGGTTGGGAAGAACTGGGTCCGACACTGTGGCAGGTTGATCCGAGCGGCACCTTTTGGGCATGGAAAGCAACCGCA
CTGGGTAAACGTAGTGATGGTAGCCGTACCTTTCTGGAACGTCGTTATAGCGAAGATCAGAGCGTTGATGATGCAATTCATACCGCAAT
TAGCACCCTGAAAGAAGGTTTTGACGGCCAGCTGACCGCAGAACTGATTGAAATTGGTGTTGTTGATGAAACCCGTAAATTTCGTACCC
TGAGCACCGCAGAAATCCGCGATTTTCTGACCGAAGTT**TAA**GAATTCAGAGGATCATAATCAGCCATACCACATTTGTAGAGGTTTTAC
TTGCTTTAAAAAACCTCCCACACCTCCCCCTGAACCTGAAACATAAAATGAATGCAATTGTTGTTGTTAACTTGTTTATTGCAGCTTAT
AATGGTTACAAATAAAGCAATAGCATCACAAATTTCACAAATAAAGCATTTTTTTCACTGCATTCTAGTTGTGGTTTGTCCAAACTCAT
CAATGTATCTTATCATGTCTGGATCTGATCACTGCTTGAGCCTAGAAGATCCGGCTGCTAACAAAGCCCGAAAGGAAGCTGAGTTGGCT
GCTGCCACCGCTGAGCAATAACTATCATAACCCCTAGGGTATACCCATCTAAGGTAGCGAGTTTAAACACTAGTATCGATTCGCGACCT
ACTCCGGAATATTAATAG**ATCATGGAGATAATTAAAATGATAACCATCTCGCAAATAAATAAGTATTTTACTGTTTTCGTAACAGTTTT**
GTAATAAAAAAACCTATAAATATTCCGGATTATTCATACCGTCCCACCATCGGGCGCCCGCGGGCCACC**ATG**ACCTATCGTTATGATGC
AGGCACCACCACCTTTAGCAGTGATGGTCGTATTCTGCAGGTTGAATATGCAATTCAGAGCATTAATCAGGCAGGCACCGCAATTGGTG
TTCAGTTTACCAATGGTGTTGTTCTGGCAGCCGAAAAGAAAAATACCGGTCGTCTGGTTGATTACCTGTTTCCTGAGAAAATGGCCAAA
ATTGATGGTCATATTGTTACCGCAGTTGCAGGTCTGACCGCAGATGCAAATACCCTGGTTGATCTGATGCGTACCAGCGCACAGAAATA
TCTGAAAACCTATGATGAGCAGATGCCGGTTGAACAGCTGGTTCGTATGGTTTGTGATGAAAAACATAGCTATACCCAGTATGGTGGTC
TGCGTCCGTATGGTGTTAGCTTTCTGATTGCAGGTTATGATCGTCATAAAGGTTGTCAGCTGTATCTGACCGATCCGAGCGGTAATTTT
GGTGGTTGGAAAGCAACCGCCATTGGTGAAAATAATCAGACCGCACAGAGCATTCTGAAAAGCCAGTATAAAGATAATATGACCGCAAC
CGAAGCAATGGATCTGACCGTTAAAGTTCTGTGTAAAACCCTGGATAGCACCAGCCTGAGCGCAGATAAACTGGAATTTGCAGTTCTGC
AGTTTCGCGAAGAATATGGTCCGAAAGTGCGTATTCTGACCACCAGTGAAGTTGATACCCTGATGAAACGTTATGAGGAAACCATTAAA
AAGTCAGCCGAGGAAAAAGAA**TAA**GGTACCAGAGGATCATAATCAGCCATACCACATTTGTAGAGGTTTTACTTGCTTTAAAAAACCTC
CCACACCTCCCCCTGAACCTGAAACATAAAATGAATGCAATTGTTGTTGTTAACTTGTTTATTGCAGCTTATAATGGTTACAAATAAAG
CAATAGCATCACAAATTTCACAAATAAAGCATTTTTTTCACTGCATTCTAGTTGTGGTTTGTCCAAACTCATCAATGTATCTTATCATG
TCTGGATCTGATCACTGCTTGAGCCTAGAAGATCCGGCTGCTAACAAAGCCCGAAAGGAAGCTGAGTTGGCTGCTGCCACCGCTGAGCA
ATAACTATCATAACCCCTAGGGTATACCCATCTAAGGTAGCGAGTTTAAACACTAGTATCGATTCGCGACCTACTCCGGAATATTAATA
G**ATCATGGAGATAATTAAAATGATAACCATCTCGCAAATAAATAAGTATTTTACTGTTTTCGTAACAGTTTTGTAATAAAAAAACCTAT**
AAATATTCCGGATTATTCATACCGTCCCACCATCGGGCGCCTCGAGGCCACC**ATG**AGCGATTATACCCGTAGCATTACCCGTTTTAGTC
CGGATGGTCGTCTGTTTCAGATTGATCATGCACATGCAGCAGTTCAGCGTGGCACCACCGTTGTTGCAACCCGTAGTAAAGATATGATT
GTTATTGCCGTTGAGAAAACCGCAGTTGCAAAACTGCAAGATCCGCATACCTTTAGCAAAATTTGTAGCCTGGATAAACATGTGATGTG
TGCATTTGCAGGTCTGCATGCCGATGCACGTCGCCTGATTCAGAGCGGTCAGCGTCAGTGTCAGAGCCATCGTCTGACCTATGAAGATC
CGATTAGCATTGAAAACATTGCCCGTTATATTGCAACCCTGCAACTGAAAAATACCCAGAGCGGTGGTGCACGTCCGTATGGTGTTAGC
ACCCTGATTTGTGGTTTTGATGATATGACCAGCCAGCCGCATATTTATGAAACCCTGCCGAGCGGCACCTATGCAGAATGGAAAGCACG
TACCATTGGTCGTCATGATCAGACCGTTATGGAATATCTGGAAAAACACTACAAAGACGATATGACCGATGAAGAAGCACAGAAACTGG
CAATTGGTGCACTGCTGGAAGTTGTTGAAAATGGTAGCAAAAATCTGGAAGTGGCCTATATGAAACGTGGTGGTACAATGGAAATTATG
GCCGAAGAGGTTCTGGATGCACTGATTGAAAGCACCAAAGCAAAA**TAA**ACGCGTAGAGGATCATAATCAGCCATACCACATTTGTAGAG
GTTTTACTTGCTTTAAAAAACCTCCCACACCTCCCCCTGAACCTGAAACATAAAATGAATGCAATTGTTGTTGTTAACTTGTTTATTGC
AGCTTATAATGGTTACAAATAAAGCAATAGCATCACAAATTTCACAAATAAAGCATTTTTTTCACTGCATTCTAGTTGTGGTTTGTCCA
AACTCATCAATGTATCTTATCATGTCTGGATCTGATCACTGCTTGAGCCTAGAAGATCCGGCTGCTAACAAAGCCCGAAAGGAAGCTGA
GTTGGCTGCTGCCACCGCTGAGCAATAACTATCATAACCCCTAGGGTATACCCATCTAAGGTAGCGAGTTTAAACACTAGTATCGATTC
GCGACCTACTCCGGAATATTAATAG**ATCATGGAGATAATTAAAATGATAACCATCTCGCAAATAAATAAGTATTTTACTGTTTTCGTAA**
CAGTTTTGTAATAAAAAAACCTATAAATATTCCGGATTATTCATACCGTCCCACCATCGGGCGCAAGCTTGCCACC**ATG**TTTAATAGCG
GCAGCGAATATGATCGCAATGTGAATACCTTTAGTCCGGATGGTCGTCTGCTGCAAGTTGAATATGCAATTGAAGCAGTTAAACTGGGT
AGCAGCGCAGTTGCAATTCTGTGTCCGGAAGGTGTTATTTTTGCAGTTGAAAAACGTCTGAGCAGCCAGCTGCTGATTGCAAGCAGCGT
TGAAAAAGTTTATGCCATCGATGATCATGTTGGTGTTGTTATGGCAGGTCTGGCAGCAGATGGTCGTACAATGGTTGAACACATGCGTG
TTGAAGCACAGAATCATCGTTTTAGCTTTGATGAACCGATTGGCATTAAAGCAGTTACCCAGAGCGTTTGTGATCTGGCACTGGCATTT
GGTGAAGGTCGTCGTAAGAAAGGTGATGGTCAGATGAGCCGTCCGTTTGGCACCGCACTGCTGGTTGCAGGTATTGAAAATGGTAAATG
TCACCTGTTTCATACCGATCCGAGCGGCACCTATACCGAATGTCGTGCACGTGCCATTGGTGGTGGTAGCGAAGGTGCCGAAGCACTGC
TGCGTGATCTGTATAAAGATGGTATGACCCTGCATGAAGCAGAGGATCTGGCCCTGAGCACCCTGCGTCAGGTTATTCAAGAAAAACTG
AATGAGAACAATGTGGAAGTTGCATGTGCACGTGTTAGCACCGGTAAATTTGAAATCTATACCAGCGAACAGCGCCAAGAAATTGTTGC
CCGTCTGCCTCCGCCTATTATTCCGGAA**TAA**GTCGACAGAGGATCATAATCAGCCATACCACATTTGTAGAGGTTTTACTTGCTTTAAA
AAACCTCCCACACCTCCCCCTGAACCTGAAACATAAAATGAATGCAATTGTTGTTGTTAACTTGTTTATTGCAGCTTATAATGGTTACA

AATAAAGCAATAGCATCACAAATTTCACAAATAAAGCATTTTTTTCACTGCATTCTAGTTGTGGTTTGTCCAAACTCATCAATGTATCT
TATCATGTCTGGATCTGATCACTGCTTGAGCCTAGAAGATCCGGCTGCTAACAAAGCCCGAAAGGAAGCTGAGTTGGCTGCTGCCACCG
CTGAGCAATAACTATCATAACCCCTAGGGTATACCCATCTAAGGTAGCGAGTTTAAACACTAGTATCGATTCGCGACCTACTCCGGAAT
ATTAATAG**ATCATGGAGATAATTAAAATGATAACCATCTCGCAAATAAATAAGTATTTTACTGTTTTCGTAACAGTTTTGTAATAAAAA**
AACCTATAAATATTCCGGATTATTCATACCGTCCCACCATCGGGCGCCCCGGGGCCACC**ATG**TTCCGCAGCAAATATGATGAAAATGCC
ACCACCTTTAGTCCGGAAGGTCGTATTCTGCAAGTTGAAAATGCAATGAAAGCAGTTCAGCAGGGTATGCCGACCGTTGGTCTGAAAAG
CAAAACCCATGCAGTTATTGCCGGTGTTATGCATAGCCCGAGCGAATTTAGCAGCCATCAGCCGAAAATCTTTAAAATCGATCAGCATA
TTGGTGTGGCAATTAGCGGTCTGACCGCAGATGGTCGTGGTCTGTGTAAATTTCTGCGTAATGAATGTCTGCATCACACCTTTTGTTTT
GGCACCGAAATTCGTGTTGCCGATCTGGCAGATACCGTTGCACTGCAAAGCCAGAAAAAGACCAGCAAAGTTGGTAAACGTCCGTATGG
TGTTGGTCTGCTGATGATTGGTGCGGGTGTTGATGGTCCGCGTCTGTTTGAAACCTGTCCGAGCGGTCAGCATTGGGAATATAATGCAC
AGGCAATTGGTCGTCGTGCCCAGGCAGCAAAAACCTATCTGGAAACCAATCTGAATGAATTTCCGGATTGTACCCGTGATCAACTGATT
CGTCATGCACTGCGTGCACTGAATGATTGTAAAAGCCGTGAAAGCGATAGCCTGGAAGCAATTGCACTGGGTGTTGTTGGTATTGATGA
ACCGTTTACCATTCTGGAAGGTCCGGAATTACAGAAATATATCGAT**TAA**AGGCCTAGAGGATCATAATCAGCCATACCACATTTGTAGA
GGTTTTACTTGCTTTAAAAAACCTCCCACACCTCCCCCTGAACCTGAAACATAAAATGAATGCAATTGTTGTTGTTAACTTGTTTATTG
CAGCTTATAATGGTTACAAATAAAGCAATAGCATCACAAATTTCACAAATAAAGCATTTTTTTCACTGCATTCTAGTTGTGGTTTGTCC
AAACTCATCAATGTATCTTATCATGTCTGGATCTGATCACTGCTTGAGCCTAGAAGATCCGGCTGCTAACAAAGCCCGAAAGGAAGCTG
AGTTGGCTGCTGCCACCGCTGAGCAATAACTATCATAACCCCTAGGGTATACCCATCTAAGGTAGCGAGTTTAAACACTAGTATCGATT
CGCGACCTACTCCGGAATATTAATAG**ATCATGGAGATAATTAAAATGATAACCATCTCGCAAATAAATAAGTATTTTACTGTTTTCGTA**
ACAGTTTTGTAATAAAAAAACCTATAAATATTCCGGATTATTCATACCGTCCCACCATCGGGCGCCATATGGCCACC**ATG**AGCGGTGCA
GGTAGCGGTTATGATTTTAATCCGATTACCTTTAGTCCGGATGGTCGTCAGTTTCAGGTTGAATATGCAACCAAAGCCGTTGAAAAAGA
TAGCCTGGCACTGGGTGTTAAATGCAAAGATGGTATTCTGCTGGCAGCCGAGAAAAATCTGACCAGCACACTGCTGACCCCTGGTGGTA
ATCCGCGTATCTTTTGGATTAATGATAGCATTGCATGTGCCACCATTGGTCATCGTCCGGATTGTTATAGCATTGTTGAACAGAGCCGT
AATCGTGCAGAAACCTTTACCAGCAATTTTGGCATCAAAATTACCGTTCCGCAGCTGGCAAGCGAAGTTAGCCAGCAGTTTCATCTGGC
ACATTATTATCAGGCATATCGTCCGTTTGGTTGTACCGTTATTTTTGCCAGCTATAAAGATGATGCCCTGTATGCAATTGAACCGAGCG
GTGCCTTTTATGGTTATTTTGCAAGCTGCTTTGGCAAGAATAGCAATCTGGCACGTGCAGAATTACAGAAAACCGAATGGAAAAATATC
ACCGTTCGTGAAGCAGTTCCGGAAGTTGCACGTATTATCAAAAGCCTGCATGAAAGCCAGTTTAAAAAGTGGGAAATCGAAATGTTTTG
GCTGTGCGAAGAAACCAATGGTCGTCCGCAGAAAGTGCCGGAAGATGTTTTTCAGAGCCGTTTTGTTAATGAAAACCCGCAGAAT**TAA***g*
cggccgc

Insert ***BamHI_β1_β2_β3_β4_β5_β6_β7+tag_ NotI** - sequences encoding Kozak sequence, **start** and **stop** codon, gene, **polyhedrin promoter**, SV40 polyA tail and twin-strep tag.are highlighted.*

ggatccGCCACC**ATG**AGCGAATATCAGTTTCCGAAAGAAAGCATGGGTACAACCCTGCTGGCAATTCAGTGTACCGATGGTGTTGTTAT
GGCAAGCGATAGCCGTACCAGCAGCGGTAGCTTTATTCCGAATCGTGCAACCAACAAAATTACCGAAATTCAGCCGAAAATCTTTGCAG
CACGTTGTGGTAATGCAGCAGATACCCAGTTTCTGGCACGTGCAGTTAAAAACTATCTGAATGCACTGAACATCACCCGTGAAAATACC
GATGATAGCACCATTCTGGTTGCCAGCAATGTTATTCGTAGCCTGATTGTTCGTTATCGTCAGTATCTGAGTGCCGGTGTTATTGTTGG
TGGTTGGGATAGCGCAGGTCCGCAGGTTTATAGCATTGAAGTTAGCGGTATGGCCATCAAAAAGAAAATTGCAAGCAATGGTAGCGGCA
GCACCTATATTCAGGCATATATTGATCAGAACTATCGCGAGGATATGACAATGGAAGAAGCAACCAAATTTGCAATTGCCGCAGTTACC
GGTGCAATTATTCGTGATGGTAGCAGCGGTGGTGTTGTGAATATTGTTCAGATTAATGCAGATGGTGCCAAACGTATGACCGTTCGTCC
GGCACAGCAGCCGTTTAACTATGATATTGTTAAAGGT**TAA**AGATCTAGAGGATCATAATCAGCCATACCACATTTGTAGAGGTTTTACT
TGCTTTAAAAAACCTCCCACACCTCCCCCTGAACCTGAAACATAAAATGAATGCAATTGTTGTTGTT**AACTTGTTTATTGCAGCTTATA**
ATGGTTACAAATAAAGCAATAGCATCACAAATTTCACAAATAAAGCATTTTTTTCACTGCATTCTAGTTGTGGTTTGTCCAAACTCATC
AATGTATCTTATCATGTCTGGATCTGATCACTGCTTGAGCCTAGAAGATCCGGCTGCTAACAAAGCCCGAAAGGAAGCTGAGTTGGCTG
CTGCCACCGCTGAGCAATAACTATCATAACCCCTAGGGTATACCCATCTAAGGTAGCGAGTTTAAACACTAGTATCGATTCGCGACCTA
CTCCGGAATATTAATAG**ATCATGGAGATAATTAAAATGATAACCATCTCGCAAATAAATAAGTATTTTACTGTTTTCGTAACAGTTTTG**
TAATAAAAAAACCTATAAATATTCCGGATTATTCATACCGTCCCACCATCGGGCGCGAGCTCGCCACC**ATG**GAAGCAGGTCTGGGTTTT
GATTTTAGCAATTATGCCCGTAACAAAAGCCTGGAACCGAAACTGGGTAAACCGCAGCTGCTGAGCACCGGCACCACCATTGCAGCAGC
AATTTTTGATGGTGGTGTTGTTCTGGGTGCAGATACCCGTGCAACCGCAGGTCCGATTGTTGCAGTTAAAGATGAAATGAAACTGCACT
ACATCAGCGATAACATTTGGGTTTGTGGTGCAGGTATTGCAGCCGATAATGATAACATTAATGCAGTGATTAGCGCCAAACTGCGTCTG
TTTCAGATGAATACCGGTCTGCAACCGCGTGTTGATCAGTGTACCAATATTCTGGCAAGTCGCCTGTTTCAGTATATGGGTTATATTCA
GGCAGCACTGATTGTTGGTGGTATTGATTTTCAGGGTCCGCAGGTTTATCAGGTTGCACCGCATGGTAGCTTTAGCAAACAGCCGTTTA
TTGCACAAGGTAGCGGTAGCCTGGCAGCAATTAGCGTTCTGGAAAATCGTTGGCATAACAAAATGAACGAACACGACTGTATGGAAATG
GTTGCAGATGCAATTTATGCCGGTATTACCAATGATTTAGGTTCAGGTAGCCATGTTAATCTGTGCGTGATTAAACGTGAAAACCCGGA
AGATAAACAGAGCAAAGTGATCTACACCTTCTATAAAGATTATCGCGTGCCGCATGAAAACGATCGTAATTTTCGTCTGGAACCGCAGA
TCAATAACATTGATGTGGAAGTGATTAAAACCACCGAACGTCCGCTGACACTGCCGGATGTTCATCTGGAAATTCTGGATGATGCACCG
GCA**TAA**GAATTCAGAGGATCATAATCAGCCATACCACATTTGTAGAGGTTTTACTTGCTTTAAAAAACCTCCCACACCTCCCCCTGAAC
CTGAAACATAAAATGAATGCAATTGTTGTTGTT**AACTTGTTTATTGCAGCTTATAATGGTTACAAATAAAGCAATAGCATCACAAATTT**
CACAAATAAAGCATTTTTTTCACTGCATTCTAGTTGTGGTTTGTCCAAACTCATCAATGTATCTTATCATGTCTGGATCTGATCACTGC
TTGAGCCTAGAAGATCCGGCTGCTAACAAAGCCCGAAAGGAAGCTGAGTTGGCTGCTGCCACCGCTGAGCAATAACTATCATAACCCCT
AGGGTATACCCATCTAAGGTAGCGAGTTTAAACACTAGTATCGATTCGCGACCTACTCCGGAATATTAATAG**ATCATGGAGATAATTAA**
AATGATAACCATCTCGCAAATAAATAAGTATTTTACTGTTTTCGTAACAGTTTTGTAATAAAAAAACCTATAAATATTCCGGATTATTC
ATACCGTCCCACCATCGGGCGCCCGCGGGCCACC**ATG**AGCGATATCAGCACCTATAATGGTAGCTGTGTTCTGGCAATGGCAGGCGATC
ATTGTGTTGCAATTGCCAGCGATCGTCGTCTGGGTGTTAATATGCTGACCGTTAGCAAAGATTTCAAACGCATCTTTCAGATCAACGAC
CGCATTTATCTGGGTTTAGCAGGTCTGGCAACCGATGTTCTGACCGTTCGTGAACAGCTGCGTTTTGATGTTAATCTGCTGGAACTGCG
TGAAGAACGTCCGATTGATCCGAAAAAGTTTATGAATCTGGTTAAAAGCACCCTGTACGAAAAACGCTTTAGCCCGTTCTTTGTTACAC
CGGTTATTGCAGGTCTGCTGCCGGAAACCAATGAACCGTATCTGGCAGCAAGCGATAGCATTGGTGCATTTGCATTTCCGAAAGATTTT
GCAGTTGCAGGCACCTGTGAAGAAAGCCTGTATGGTATTTGTGAAAGCGCATGGCGTCCGAATATGAATCCGGATGAACTGTTTGAATG
TACCGCCAAATGTCTGATTGCAGCAGTTGAACGTGATAGCATTAGCGGTTGGGGTGGTATTGTTTATATCATCACCCAGGATAAAGTGA
TCATCAAAGAAATCAAAACCCGCATGGAT**TAA**GGTACCAGAGGATCATAATCAGCCATACCACATTTGTAGAGGTTTTACTTGCTTTAA
AAAACCTCCCACACCTCCCCCTGAACCTGAAACATAAAATGAATGCAATTGTTGTTGTT**AACTTGTTTATTGCAGCTTATAATGGTTAC**
AAATAAAGCAATAGCATCACAAATTTCACAAATAAAGCATTTTTTTCACTGCATTCTAGTTGTGGTTTGTCCAAACTCATCAATGTATC

TTATCATGTCTGGATCTGATCACTGCTTGAGCCTAGAAGATCCGGCTGCTAACAAAGCCCGAAAGGAAGCTGAGTTGGCTGCTGCCACC
GCTGAGCAATAACTATCATAACCCCTAGGGTATACCCATCTAAGGTAGCGAGTTTAAACACTAGTATCGATTCGCGACCTACTCCGGAA
TATTAATAGATCATGGAGATAATTAAAATGATAACCATCTCGCAAATAAATAAGTATTTTACTGTTTTCGTAACAGTTTTGTAATAAAA
AAACCTATAAATATTCCGGATTATTCATACCGTCCCACCATCGGGCGCCTCGAGGCCACCATGCTGAGCATTGTTGGTCTGCAAGGTCC
GGATTGGGTTCTGATTGCAGCAGATAGCAGCGTTAGCAGCAGCATTATTTGTATGAGCGAAAACTATGATCGTATCGCACAGCTGGATG
ATCGTCATGCACTGGCAATGAGCGGTGAAACCGGTGATTGTCTGCAACTGAGCGAATATTTACAGGGTAATGTTGCCCTGTACAAATTT
CGTAATGGTGTTGAACTGAGCAGTGATGCCCTGGCACATTTTATTCGTCATACAATGGCAAAAGCCGTTCGTAAAAGCCCGTATGAAGT
TAATATGCTGCTGAGCGGTTATGATGGTAAACCGCATCTGTATTTCATGGATTATCTGGGCACCCTGCAAAGCATTCCGTATGGTGCAC
AGGGTTATTGTCAGTATTTTGTGATGAGCGTGTTCGATAAGCACTATAAAGAAGGTCTGACCCTGGAAGATGGTAAAGAACTGATGAAA
CTGGCACTGAACCAGATTAAACAGCGTTTTACCGTTGCACCGCATGGCTTTATTGTTAAACTGGTGGATAAAAACGGCATCACCAAAAT
CGATCTGGAATAAACGCGTAGAGGATCATAATCAGCCATACCACATTTGTAGAGGTTTTACTTGCTTTAAAAAACCTCCCACACCTCCC
CCTGAACCTGAAACATAAAATGAATGCAATTGTTGTTGTTAACTTGTTTATTGCAGCTTATAATGGTTACAAATAAAGCAATAGCATCA
CAAATTTCACAAATAAAGCATTTTTTTCACTGCATTCTAGTTGTGGTTTGTCCAAACTCATCAATGTATCTTATCATGTCTGGATCTGA
TCACTGCTTGAGCCTAGAAGATCCGGCTGCTAACAAAGCCCGAAAGGAAGCTGAGTTGGCTGCTGCCACCGCTGAGCAATAACTATCAT
AACCCCTAGGGTATACCCATCTAAGGTAGCGAGTTTAAACACTAGTATCGATTCGCGACCTACTCCGGAATATTAATAGATCATGGAGA
TAATTAAAATGATAACCATCTCGCAAATAAATAAGTATTTTACTGTTTTCGTAACAGTTTTGTAATAAAAAAACCTATAAATATTCCGG
ATTATTCATACCGTCCCACCATCGGGCGCAAGCTTGCCACCATGCAGAGCCTGTATCTGAAACCGCGTGATGAAGTTGAAAGCGAAGAA
ACCACCAAAGCACTGGAACCGGCACATGTTCCAGATCCGTGTCAGTTTGTTAAAAACCATATTAGCCTGAGCTATACCAACGAACCGGG
TAAAATGGCAGCAGTTCATGGCACCACCACACTGAGCTTTATCTATAATGGTGGTATTGTTGTTGCCGTTGATAGCCGTGCAACCGGTG
GTCAGTTTATCTTTAGCCAGACCGTTATGAAAATTCTGCCGCTGGCACCGAATATGATTGGTACAATGGCAGGCGGTGCAGCAGATTGT
CAGTATTGGCTGCGTAATCTGAGCCGTCTGATTCAGCTGCATAAATTTCGTTATCAGCAGCCGCTGACCGTTGCAGCAGCAAGCAAAAT
TCTGGTTAATGAACTGTATCGCTACAAGGGCTATAATCTGAGTATTGGTAGCATGATTTGCGGCTATGATAATACCGGTCCGCACATCT
TTTATATCGATAATCATGGTAGCCGCATTGCCGGTAAACGTTTTAGCGTTGGTAGCGGTAGCACCCATGCCTATGGTGTTCTGGATACC
TGTTATCGTGAAGATATGACCAAAGAAGAAGCCTGCGAACTGGGTCGTCGTGCCATTTATCATGCAACCTATCGTGATAGCGGTAGTGG
TGGTCGTGTTAGCGTTGTTCATATTACCCAGAATGGTGTGGAATGGATCGATAAAACCGATGTGTTTGATATGCACGACTTTAGCAAAA
CCACCTTTTAAGTCGACAGAGGATCATAATCAGCCATACCACATTTGTAGAGGTTTTACTTGCTTTAAAAAACCTCCCACACCTCCCCC
TGAACCTGAAACATAAAATGAATGCAATTGTTGTTGTTAACTTGTTTATTGCAGCTTATAATGGTTACAAATAAAGCAATAGCATCACA
AATTTCACAAATAAAGCATTTTTTTCACTGCATTCTAGTTGTGGTTTGTCCAAACTCATCAATGTATCTTATCATGTCTGGATCTGATC
ACTGCTTGAGCCTAGAAGATCCGGCTGCTAACAAAGCCCGAAAGGAAGCTGAGTTGGCTGCTGCCACCGCTGAGCAATAACTATCATAA
CCCCTAGGGTATACCCATCTAAGGTAGCGAGTTTAAACACTAGTATCGATTCGCGACCTACTCCGGAATATTAATAGATCATGGAGATA
ATTAAAATGATAACCATCTCGCAAATAAATAAGTATTTTACTGTTTTCGTAACAGTTTTGTAATAAAAAAACCTATAAATATTCCGGAT
TATTCATACCGTCCCACCATCGGGCGCCCCGGGGCCACCATGGAAGGTGAATTTCGCGAGAATAAGAAAGGTCAGTGGTCACCGTATGA
AATGCATGGTGGCACCGCAATTGGTATTTGTGGTGATGATTATGTTGTGATTGGTGCAGATACCCGTCTGAGCGTTGATTATAGCATTG
ATAGCCGTCATAAAGCCCGTATCTTTAAGATGAATAGCAACTGTATGATTAGCGCCACCGGTTTTGATGGTGATATTGATGCATTTATT
ACCCGCATGCGTAGCATTCTGCTGAATTATGAAAACCAGCACTTTCACGAAATGAGCGTGGAAAGCGTTGCACGTTGTGTTAGCAATAC
CCTGTATAGCAAACGTTTCTTCCCGTACTATATCAACATTCTGGTTGGTGGCATTAACAGCGAAGGTAAAGGTAAACTGTATGGTTATG
ATCCGGTTGGCACCATTGAGGATCTGCATTATGATAGCAATGGTAGCGGTAGCAGCCTGGCAGCACCGCTGCTGGATAGCGCATTTGGT
ACAATTCATCATAATACCCGTCCGTTTCCGGCAGTTTCACTGCAAGATGCCAAAAACATTGTTCGTGATGCAATTTGTAGCGTTACCGA
ACGTGATATCTATACCGGTGATGCACTGCAACTGTGTGTTTTTACCAAAGATGGTTTTGCCCAAGAAGAATTTCCGCTGCCTCGTCATT
AAAGGCCTAGAGGATCATAATCAGCCATACCACATTTGTAGAGGTTTTACTTGCTTTAAAAAACCTCCCACACCTCCCCCTGAACCTGA
AACATAAAATGAATGCAATTGTTGTTGTTAACTTGTTTATTGCAGCTTATAATGGTTACAAATAAAGCAATAGCATCACAAATTTCACA
AATAAAGCATTTTTTTCACTGCATTCTAGTTGTGGTTTGTCCAAACTCATCAATGTATCTTATCATGTCTGGATCTGATCACTGCTTGA
GCCTAGAAGATCCGGCTGCTAACAAAGCCCGAAAGGAAGCTGAGTTGGCTGCTGCCACCGCTGAGCAATAACTATCATAACCCCTAGGG
TATACCCATCTAAGGTAGCGAGTTTAAACACTAGTATCGATTCGCGACCTACTCCGGAATATTAATAGATCATGGAGATAATTAAAATG
ATAACCATCTCGCAAATAAATAAGTATTTTACTGTTTTCGTAACAGTTTTGTAATAAAAAAACCTATAAATATTCCGGATTATTCATAC
CGTCCCACCATCGGGCGCCATATGGCCACCATGCAGGTTATTACCGCAAGCGGTGCAATTGTTGCAGCAAAATATGATGGTGGTATTCT
GCTGGCAAGCGATCTGAGCATTACCTATGGTAGCATGTTTCGCCATAATAACGTTAGCCATTTTGTTGAAGTTGCACCGAACATTATTA
TCGGTGCCAGCGGTGAATTTGCAGATTTTCAGACCCTGATTGAAGTGATCAAAAGCGTTATTCTGCAGCAGCAGTGTAAACATAATGGT
GAATATCTGACCGCCAGCGAAGTGCATAATTACATTAAACGCTATATGTATCAGTGCCGCAGCAATATGAAACCGCTGAGCTGTAAAGT
TATTGTGGCAGGTATTAATCCGGATGGCAGCAAATTTCTGGCATGTACCGATCCGTATGGTGCAAGCTGGGAAAGCGATCACATTGGCA
CCGGTTTTGGTAAATATCTGCAGGGTCTGCAGATTGCCGATGTTGTTAATGGTAGCTTTGATGATGTGAAAAAGGGCATCACCGAAGTT
TTTCGTGCAGTTAATGCCCGTAATACCACCGCAAATGGTAAAATCGAATTTATCACCGTTACACCGCAGGGCATTAATCATCTGGCACC
GGAACAAATTGATCCGAATTGGGAAGTTGTTGAAGGCACCTGGGATCAGAGCGCTTGGAGCCACCCGCAGTTCGAAAAAGGTGGAGGTT
CTGGCGGTGGATCGGGAGGTTCAGCGTGGAGCCACCCGCAGTTCGAGAAATAA*gcggccgc*

Insert BamHI_Ump-1_NotI

*ggatcc*GCCACCATGTACGAACAGTGGATTCCGGAAGAACCGGTTGATCGTCTGCGTGAAGGTCTGCCGAATCTGCGTCATGGTAGCGT
TAATCAGCATCCGCTGGAAATTGCCATTGAAGAACGTCGTAAAACCCAGTTCAAAGACAAATTTGATGAACTGGCACTGCTGTATGGTG
AAGGTTTTGCAAATCATGAGAAGATGCTGTACAAACTGATTAAAAGCACCCGTATTGGTTTTCGCACCTATGAACGTCCGGATGATCTG
GCAATTGAAGTTTTTACCGGTGATATCGATGATGTGGATTTCAACGATATGTTTGCACCGAATGGTATGCGTGCCGATATTGAATTTGA
TCCGCATGAAATCCAAGAGAAACGCCTGAATATTGAATAA*gcggccgc*

Supplementary Figure 2. Cloning plasmids and proteasome gene sequences. a-c) Schematic representation of the cloning of the three plasmids used to generate recombinant baculoviruses. The figure was created using SnapGene® (snapgene.com). **d)** The inserted DNA sequences correspond to the seven *Tv*20S α subunits (BamHI_α1_α2_α3_α4_α5_α6_α7_NotI), seven Tv20S β subunits (BamHI_β1_β2_β3_β4_β5_β6_β7+tag _NotI) and *Tv*20S Ump-1 (BamHI_Ump-1_NotI). The accession numbers for each subunit are listed in Supplementary Table 3. Sequences are labelled as follow: *restriction enzyme cleavage site*, Kozak sequence, **start** and **stop** codon, gene, **polyhedrin promoter**, SV40 polyA tail, twin-strep tag.

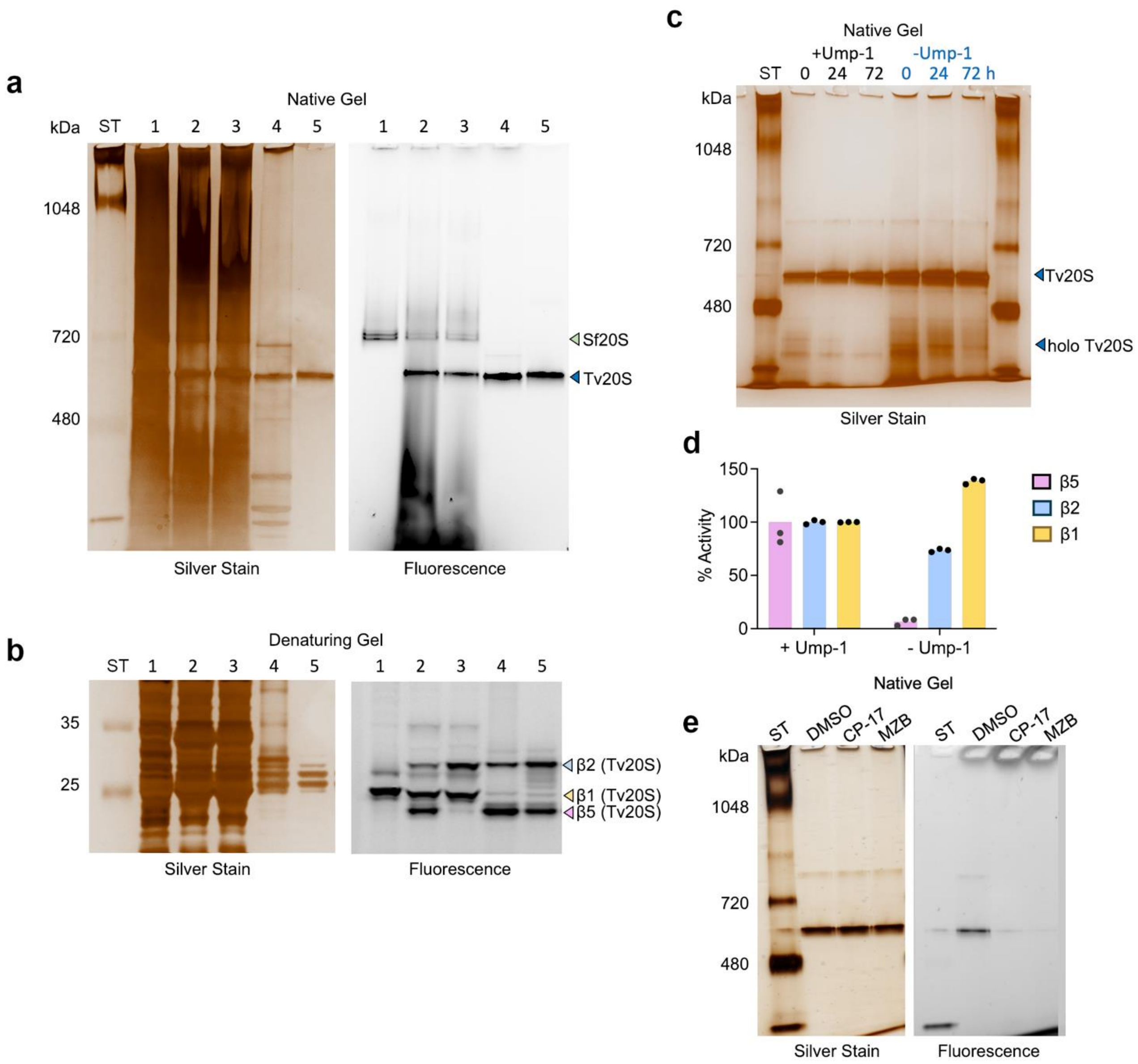

Supplementary Figure 3. Confirmation of recombinant Ump-1 function and 20S stability. Panel **a)** shows a native protein gel demonstrating Me4BodipyFL-Ahx3Leu3VS probe labelling of the proteasome. The left panel is a silver-stained gel, while the right panel is a fluorescent scan of the same gel at 470 nm excitation and 530 nm emission. Panel **b)** displays a denaturing gel with the same protein samples as panel a lane 1 represents Sf9 cell lysate, lane 2 represents Sf9 cell lysate infected with alpha, beta, and Ump-1 baculovirus, lane 3 represents Sf9 cell lysate infected with alpha and beta baculovirus, lane 4 represents native *Tv*20S, and lane 5 represents purified r*Tv*20S. Panel **c)** shows the time-dependent assembly of the 20S proteasome in the presence and absence of Ump-1, while panel **d)** shows the enzymatic activity of r*Tv*20S in the presence and absence of Ump-1. Assays were performed in technical replicates (n=3). **e)** Silver-stained gel showing the mass of r*Tv*20S is unaltered in the presence of inhibitors while the fluorescent gel shows that reduced labelling of the active sites. All gels were repeated independently two times with similar results. 'ST' stands for the molecular weight ladder.

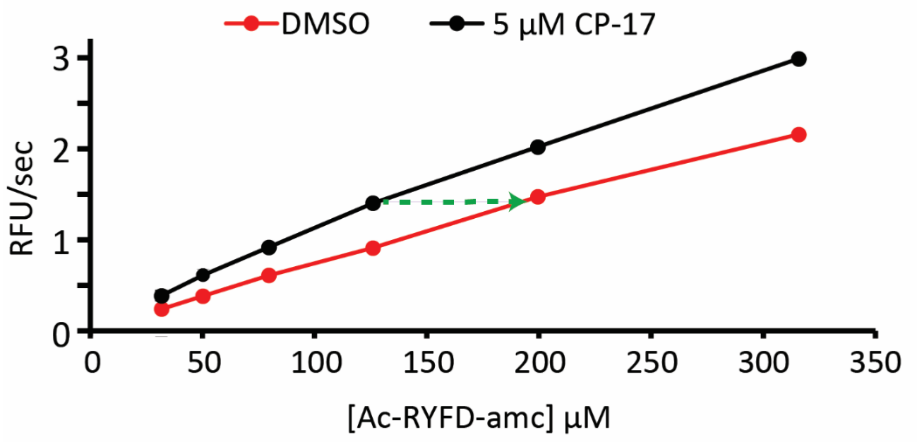

Supplementary Figure 4. Activation of β1 in the presence of CP-17. r*Tv*20S was preincubated with 5 μM of CP-17 to inactivate β5 only and then assayed with Ac-RYFD-amc. Activity was compared to a vehicle control. Assays were performed in triplicate, with each dot representing the mean of 3 technical replicates. The green arrow shows an example of the apparent increase in substrate concentration (from 126 μM to 200 μM) within the proteasome core in the presence of CP-17.

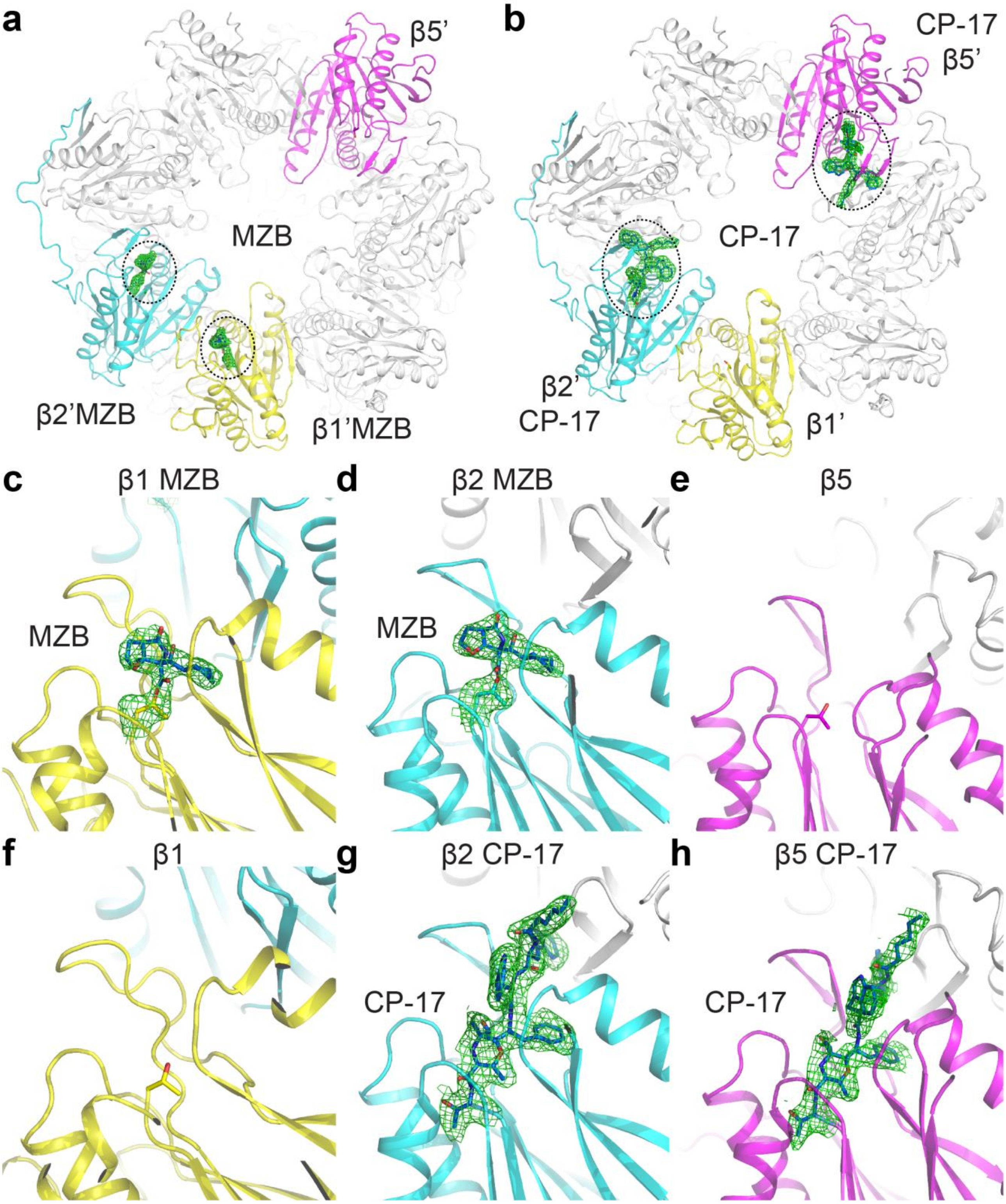

Supplementary Figure 5. Structural comparison of cryo-EM maps of the inhibitor molecules MZB and CP-17 covalently bound in the active sites of *Tv*20S

The top views of both *Tv*20S structures with **a)** MZB and **b)** CP-17 inhibitors (both shown as blue sticks). Cryo-EM Maps are carved around inhibitors for clarity as green mesh at sigma 4. Detailed views on the active sites are shown as cartoon and sticks for inhibitors. MZB (panels **c)**, **d)** and **e**) and CP-17 (panels **f**, **g** and) are displayed with active sites in order: β1 (panels **c**&f shown in yellow), β2 (panels **d) & g),** shown in cyan), and β (panel **e) & h)**, shown in magenta).

S

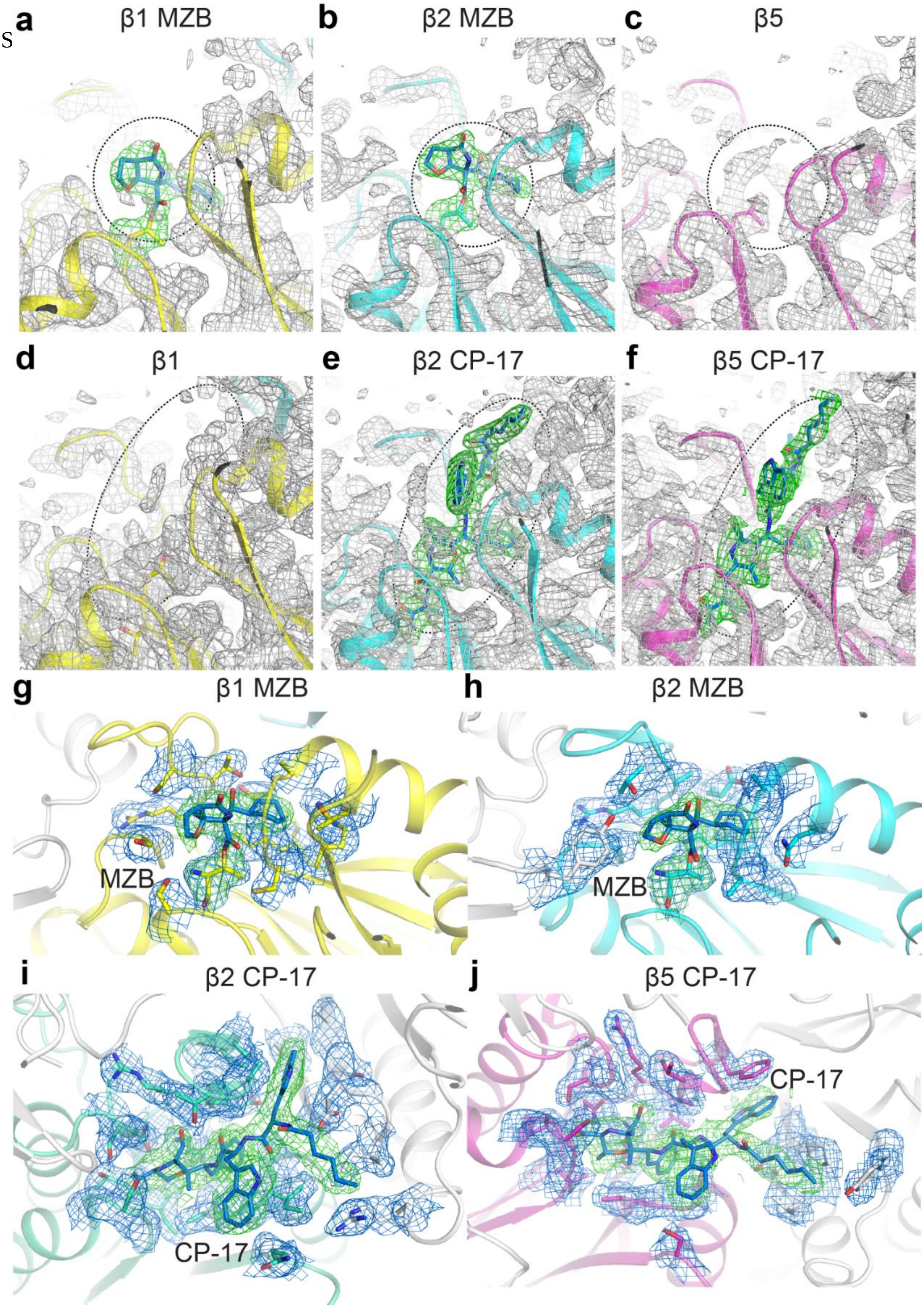

Supplementary Figure 6. Cryo-EM maps in all three active sites for both *Tv*-20S structures
Comparison of cryo-EM maps for both *Tv*20S structures with MZB (**a**,**b**,**c**,**g**,**h**) and CP-17 (**d**,**e**,**f**,**i**,**j**) inhibitors. Maps are shown as grey mesh at sigma 4 and density around inhibitors is depicted in green. Active sites are displayed in order: β1 (panels **a)** & **d)** are shown in yellow), β2 (panels **b)** & **e)**, shown in cyan), and β (panel **c)** & **f)**, shown in magenta. Panels **g)** - **j)** additionally show the density around *Tv20S* residues that form the binding site, and ligand, shown simultaneously as sticks and also cryo-EM maps at the same sigma level. The map around *Tv20S* residues is depicted in blue.

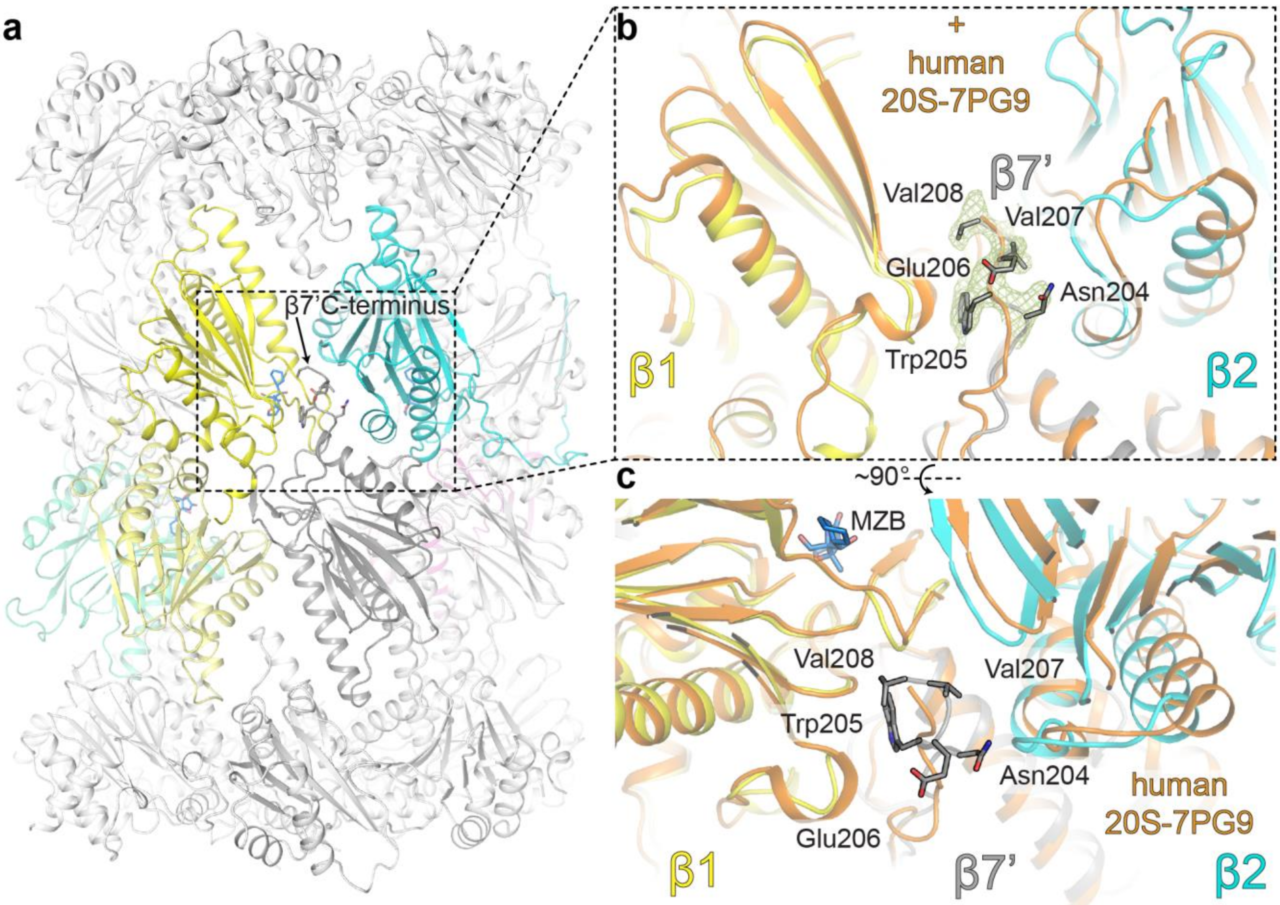

Supplementary Figure 7. C-terminal end being properly inserted between the β1 and β2. a) *Tv*20S with MZB cartoon representation where β1 is shown in yellow, β2 cyan and β7' grey with its the C-terminus marked by arrow. Panels **b)** shows local cryoEM map (green mesh) and **c)** show detailed view with an overlay with superimposed human 20S proteasome (PDB ID = 7PG9) shown in orange. The residues of β7' Asn204-Val208 of *Tv*20S shown as grey sticks inserted between β1 and β2 chains.

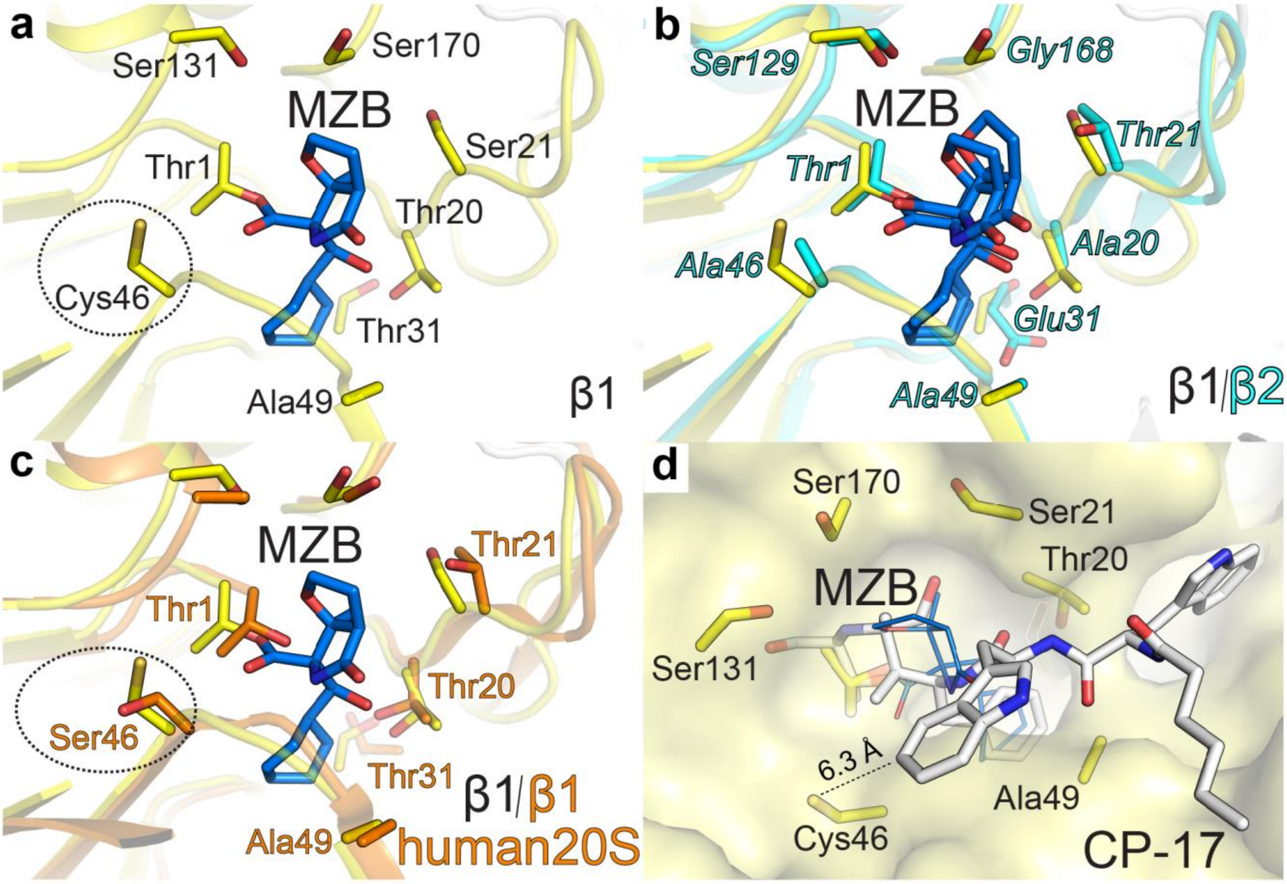

Supplementary Figure 8. Prospects for developing inhibitors that target Cys46 of the β1 subunit of *Tv*20S proteasome.

a) The active site of the β1 subunit in the *Tv*20S-MZB proteasome structure is highlighted in yellow, while MZB is shown as blue sticks. This region includes neighbouring residues, including Cys46. **b)** The structural overlay of β1 and β2 (cyan sticks) subunits of *Tv*20S-MZB proteasome. **c)** An overlay comparison is made between the β1 active site of *Tv*20S-MZB (shown in yellow, with MZB represented as blue sticks) and the human 20S structure, PDB ID = 7PG9 (shown in orange). **d)** Another overlay is presented, showcasing only the CP-17 inhibitor (depicted in white) of the β2 subunit onto the β1 active site of *Tv*20S-MZB (represented in yellow, with MZB shown as blue lines).

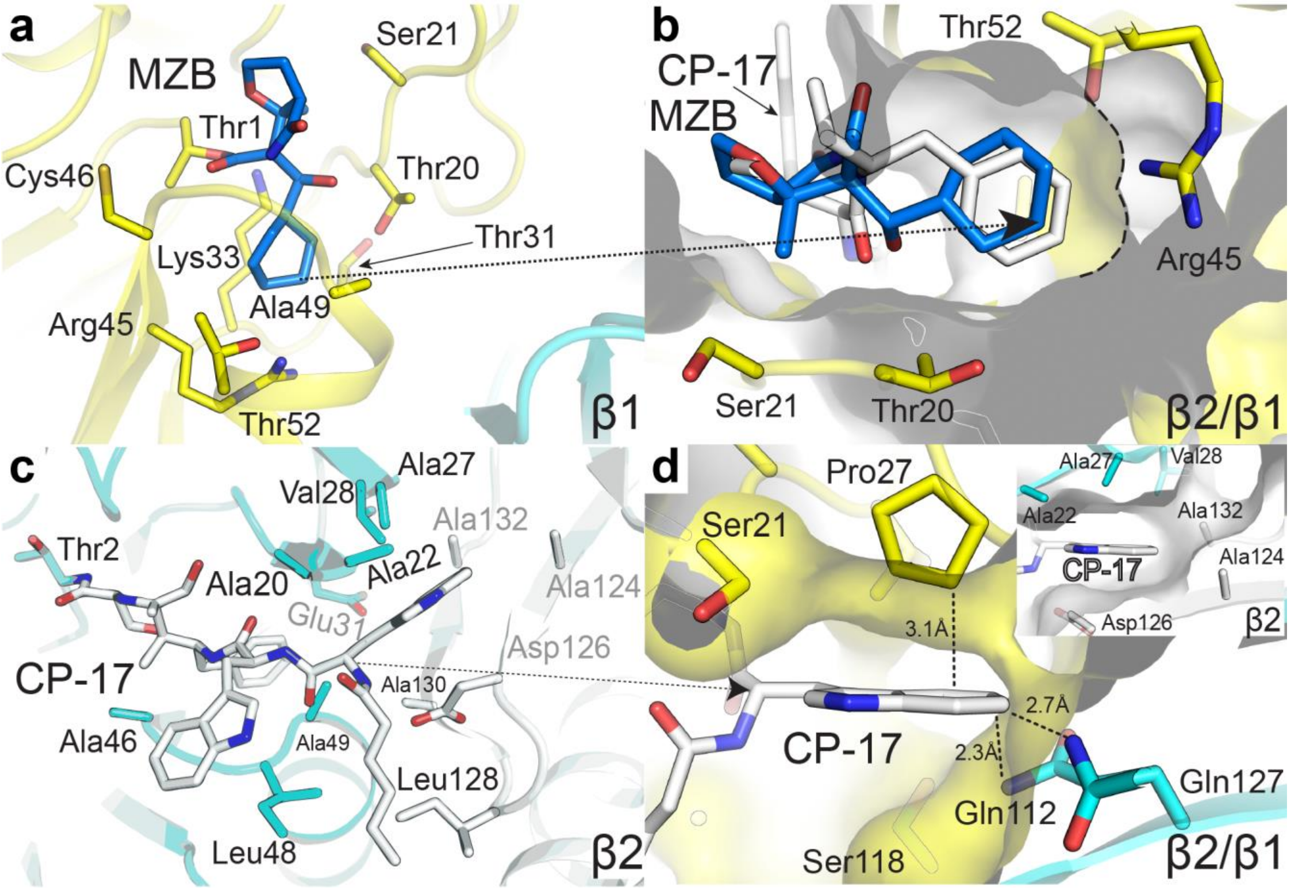

Supplementary Figure 9. Model of CP-17 "virtual" clashes within the *Tv*20S proteasome β1 active site pocket

a) The β1 active site of *Tv*20S-MZB structure (shown in yellow, MZB in blue) with neighbouring residues including Cys46. **b)** The surface of β1 with MZB (yellow and blue sticks) and β2 with and overlay of surface and CP-17 inhibitor (white and white sticks) from the β2 of *Tv*20S-CP-17 proteasome. CP-17 is shown as white sticks for clarity. **c)** Detail of β2 with CP-17 with highlighted residues mainly surrounding indole rings. **d)** The inset of the figure shows surface of β2 S3 pocket of *Tv*20S-CP-17 proteasome. The main panel shows surface of β1 site with CP-17 (white) modelled by superimposition of β2 and β1 subunits. The β1 pocket is lined with much bulkier residues such as Pro27 and polar residues Ser118, Gln112 and Gln127. Unlike in the β2 pocket lined with smaller and more hydrophobic residues Ala (22,27,124,126,132) and Val28.

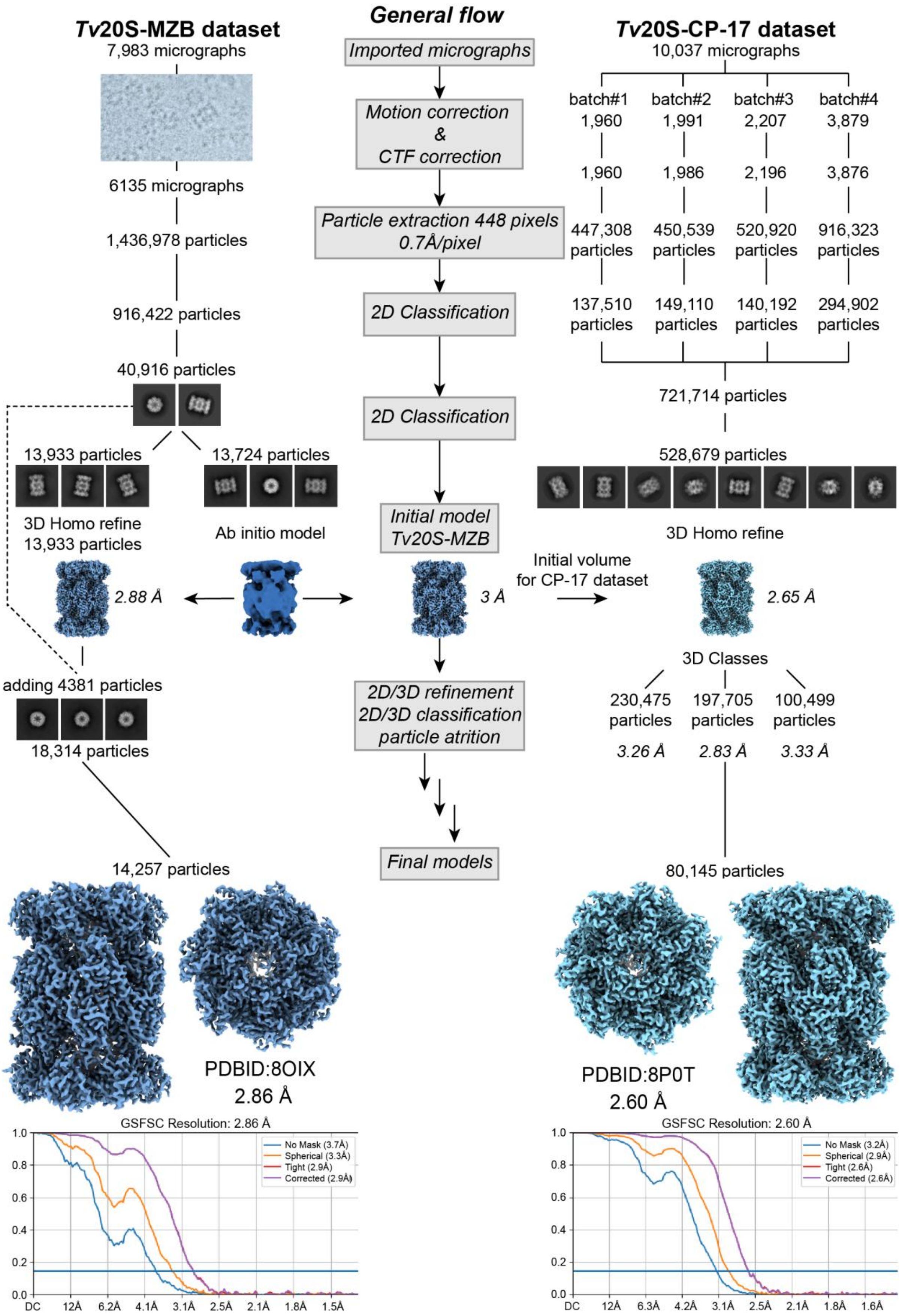

Supplementary Figure 10. Cryo-EM workflow of data processing. This image processing workflow was employed to reconstruct the *Tv*20S structures in cryo-EM. Both datasets were acquired at Titan Krios with the Falcon 4i detector under identical conditions using the same setup (refer to Supplementary Table 1 for details). The *ab initio model* served as the starting point for 3D homogeneous refinement (Homogeneous Refinement) to enhance the quality of the maps. Multiple iterations, including 3D classification and 2D classification, were

carried out in several rounds to eliminate unwanted particles, refine the resolution, and improve the maps. The unsharpened maps of the final reconstruction and the gold-standard Fourier Shell Correlation (FSC) curve using different masks are shown. The figures of the maps were generated by ChimeraX[2]. The resolution in italics corresponds to a particular Fourier Shell Correlation Cryosparc and was used to navigate the process of data analysis. The final resolution was estimated by Fourier Shell Correlation job in Cryosparc[3] when a tight mask was applied and was estimated to be 2.86 Å for 8IOX and 2.60 Å for 8P0T. An example of the *Tv*20S-MZB route of processing is as follows: 1,436,978 particles were extracted from 6135 processed images. After several rounds of 2D classification to sort out unwanted classes, only 40,916 particles remained. These were then reclassified with independent 2D classifications for the top views and the side views. Separately, classes with 13,724 particles of both views were used for an ab initio model. This initial cryo-EM map was used as a starting model for homogeneous reconstruction, where 13,933 side view particles were used. Next, 4381 particles corresponding to the top views were added in the next round. For the final model, additional particles were removed during several rounds of homogeneous reconstruction and 3D classifications. Note: clear top views of the full proteasome were assumed to be the particles with the highest contrast. Representative Cryo-EM images are shown in subsequent Supplementary Figure 11.

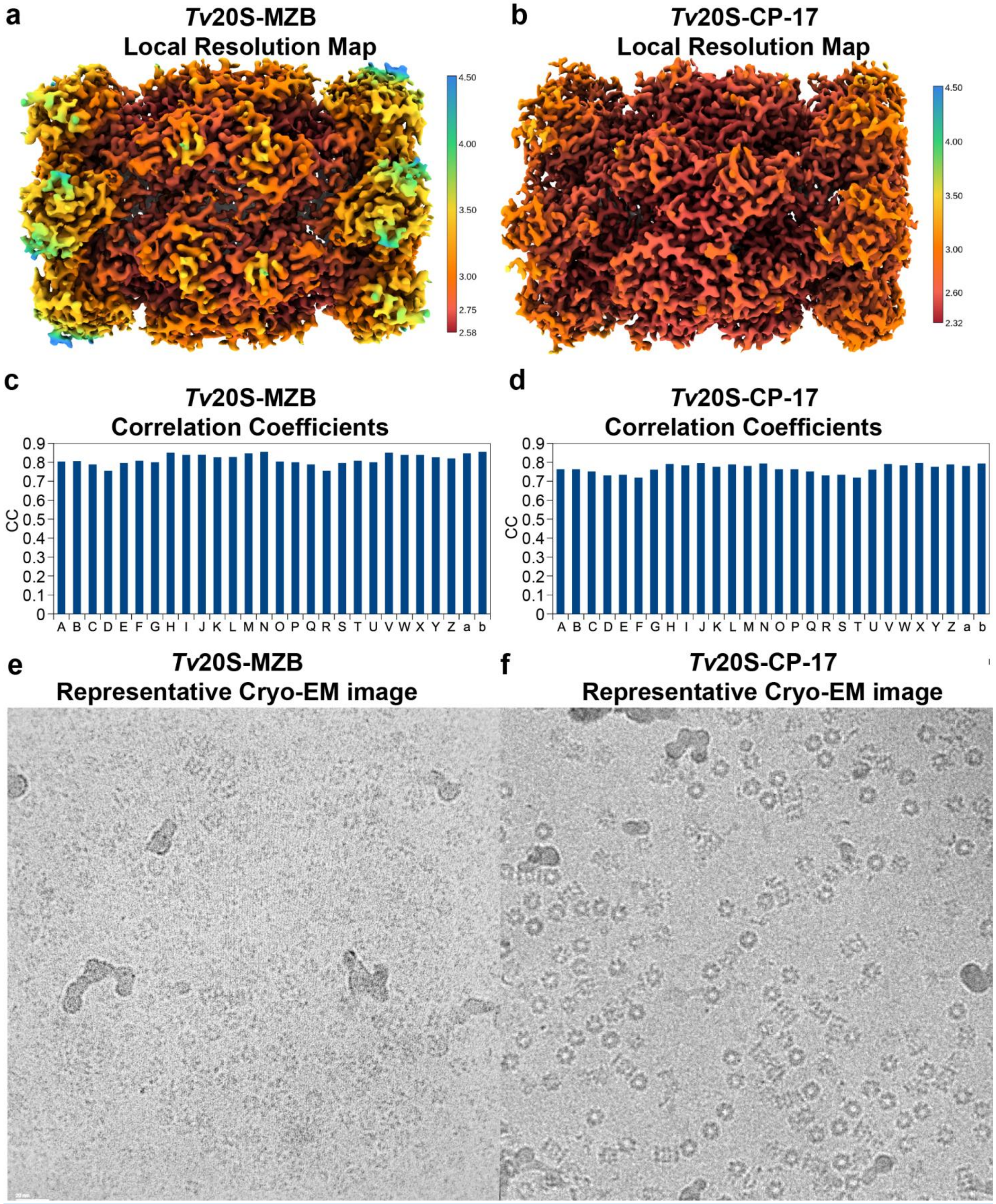

Supplementary Figure 11. Cryo-EM maps coloured according to the calculated local resolution and example of Cryo-EM images. The estimate of local resolution for both *Tv*20S structures **a)** 8OIX and **b)** 8P0T are compared. **c&d** show average map correlation coefficient for each chain. Source data are provided as a Source Data file Figures.xlsx. Panels **e&f** demonstrate the particle density and quality of Cryo-EM images for both datasets.

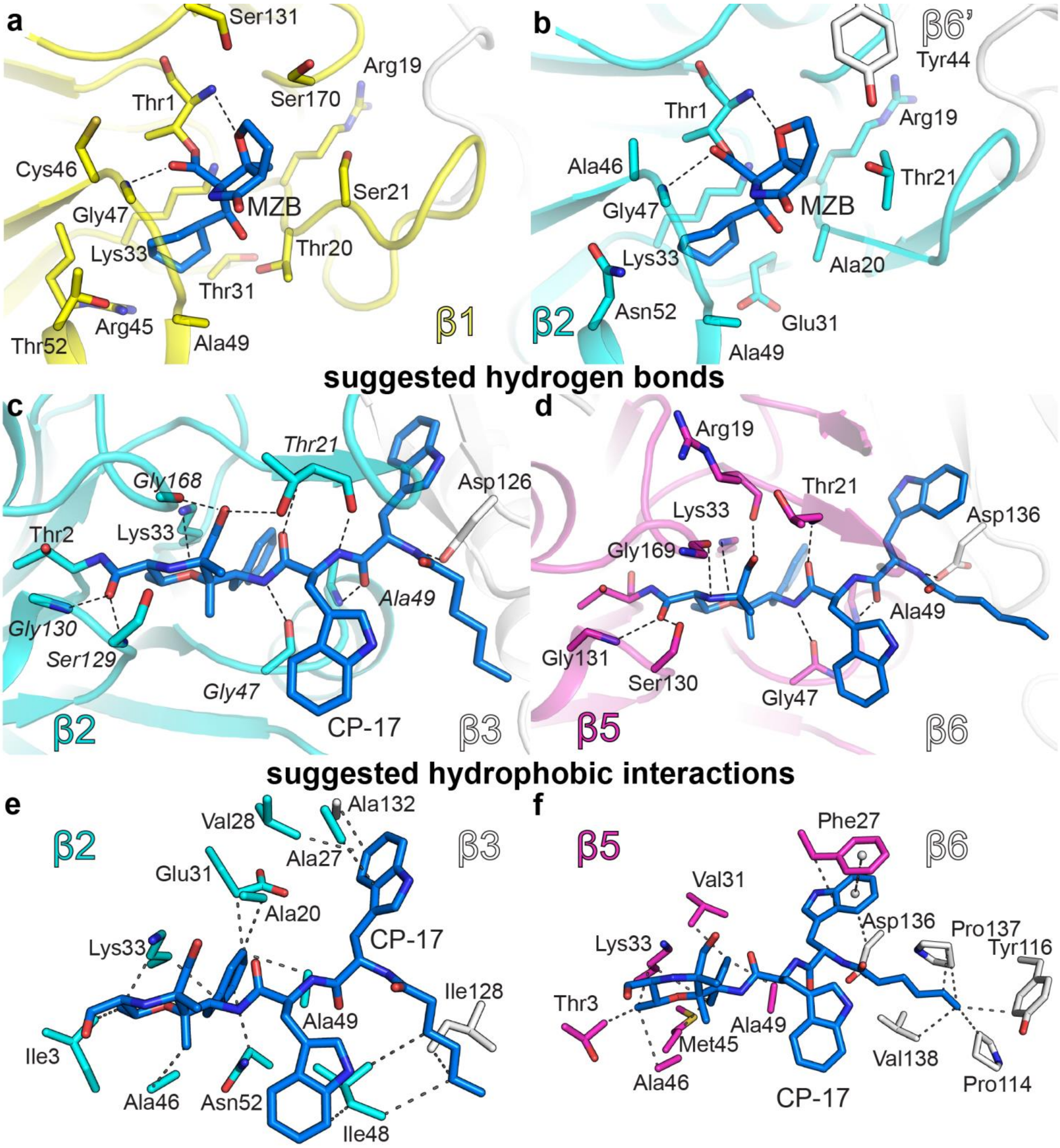

Supplementary Figure 12. Detailed interactions between small molecular inhibitors and active site pockets of *Tv*20S. The interactions between amino acid residues surrounding the active sites of *Tv*20S with the inhibitors; **a) & b)** MZB and **c)-f)** CP-17 with highlighted residues shown as sticks suggest interactions with inhibitors. Dashed lines represent hydrogen bridges in panels **a)-d)** and "hydrophobic" interactions are shown in panels **e) & f)**.

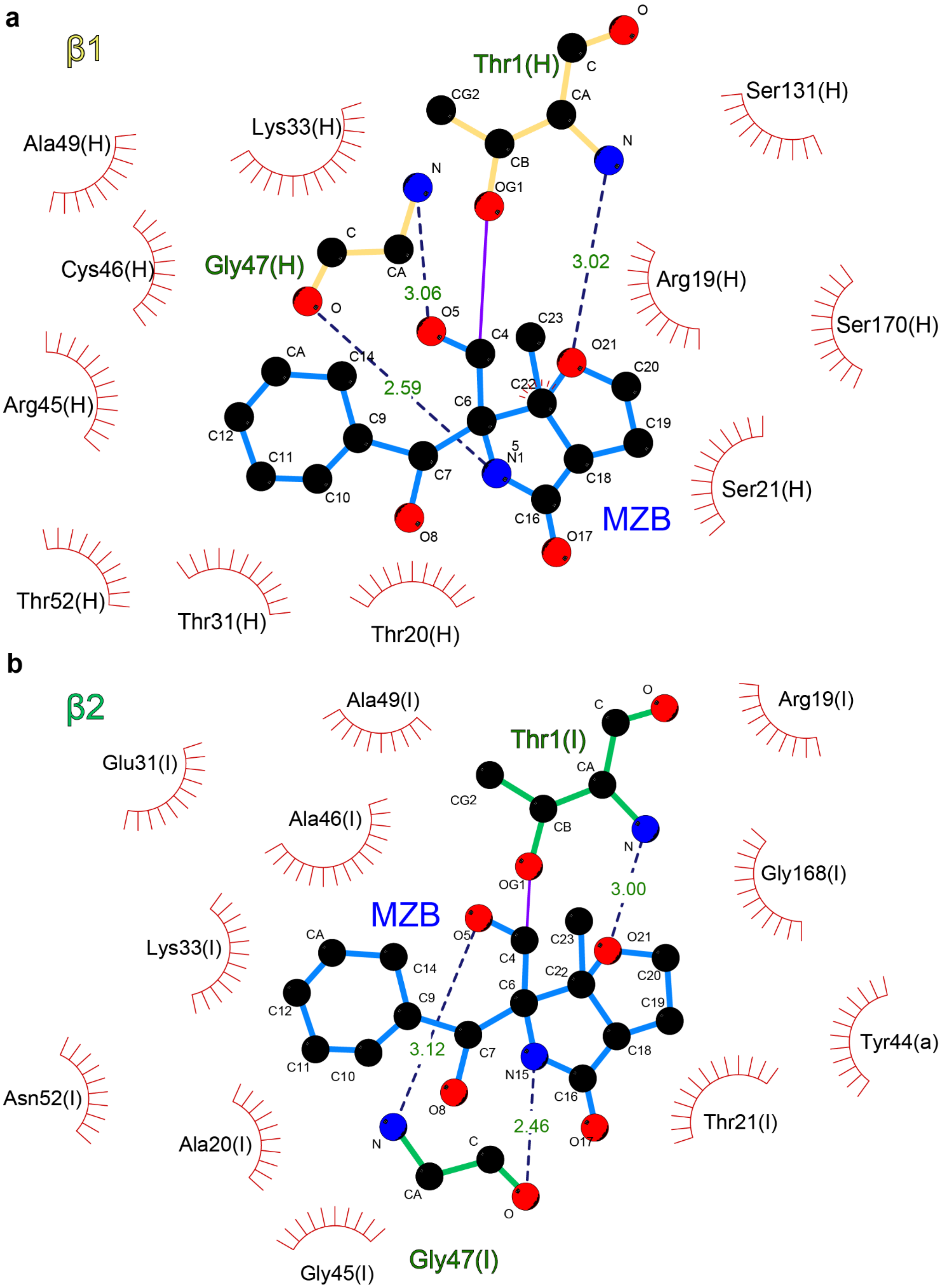

Supplementary Figure 13. MZB 2D representation of interactions between small molecular inhibitor MZB and *Tv*20S active site. a) & **b)** highlight residues in close contact with inhibitor in β1 and β2 active sites. Dashed lines represent hydrogen bridges, and "fan-like structures" demonstrate possible hydrophobic interaction.

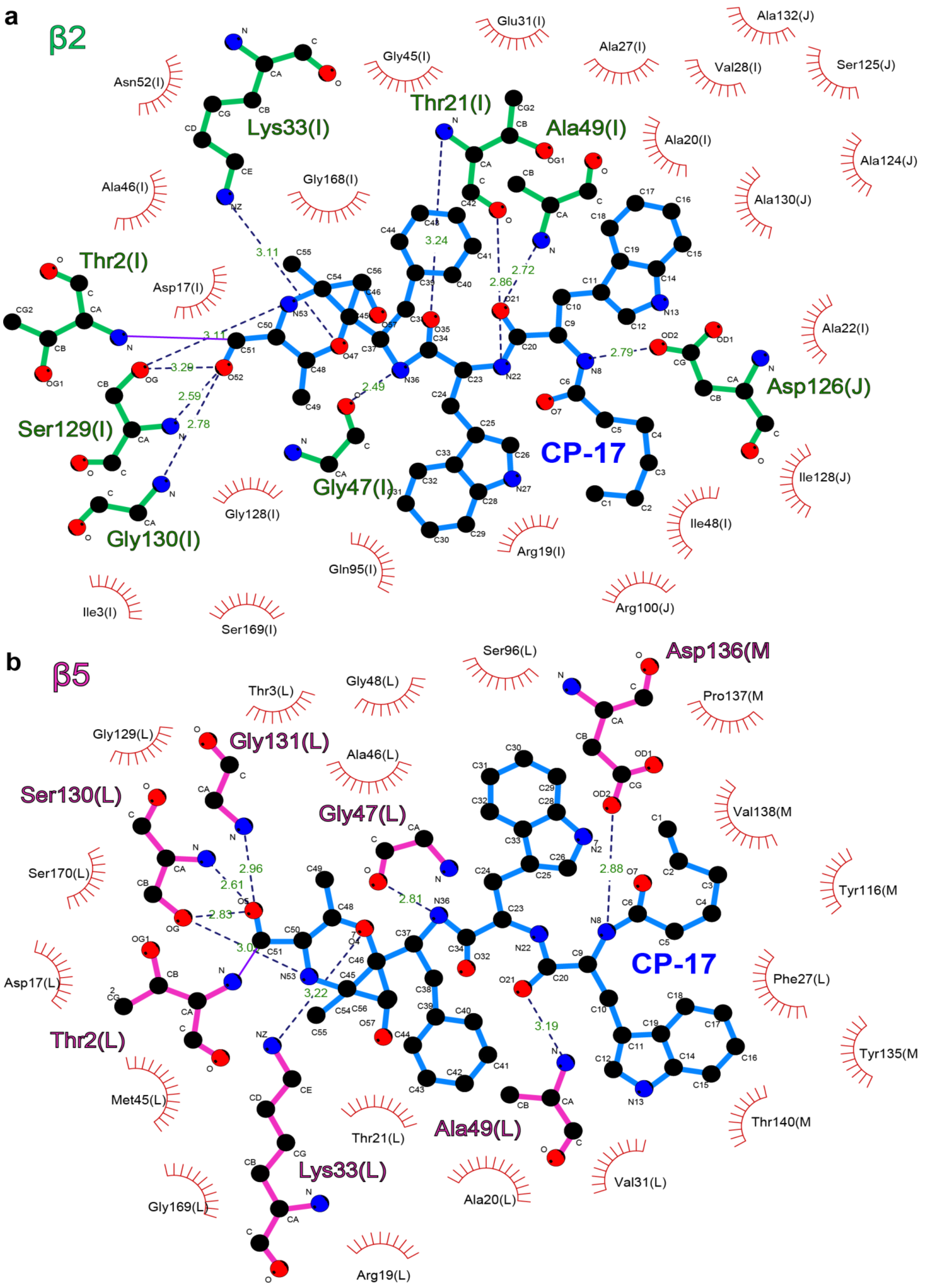

Supplementary Figure 14. The lig plot CP-17 2D representation of interactions between molecular inhibitor CP-17 and the active site of *Tv*20S. a) & b) highlight residues in close contact with inhibitor in β2 and β5 active sites. Dashed lines represent hydrogen bridges, and "fan-like structures" demonstrate possible hydrophobic interaction.

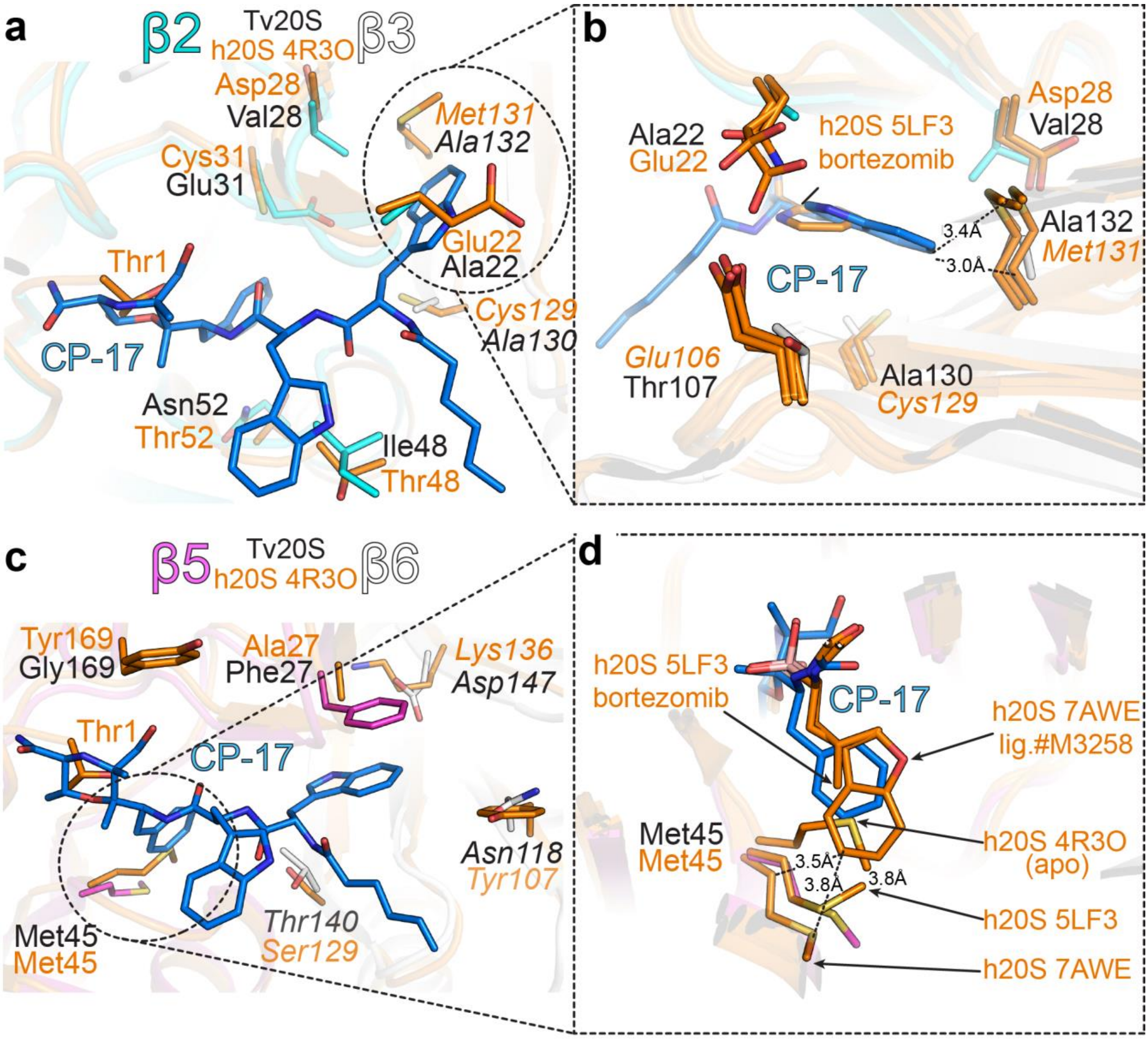

Supplementary Figure 15. The main differences of *Tv*20S and potential clashes CP-17 in human 20S proteasome β2 and β5 active site pockets. Structural superimposition of *Tv*20S-CP-17 (8P0T) with human 20S (PDBID = 4R3O, and 5LF3 with 7AWE in right hand panels). **a**) The rotamer of human 20S (PDB ID = 4R3O) Met131 (β3) is relatively close and bulky compared to Ala of *Tv*20S, **b**) another view to the β2 active site besides 4R3O structure with the overlay of additional two structures 5LF3, 7AWE shows potential clash between CP-17 and Met131. Additionally, the Glu106 (β3) and Glu22 (β2) of human 20S suggest additional site of steric hindrance for CP-17 Panels **c**) & **d**) show similar contact of possible clash yet the rotamers in different structures such as 7AWE show deflection from the possible clash. Human 20S proteasome is shown in orange.

Supplementary Table 1. Structural alignment of 20S proteasome *Tv*20-CP1 7ZYJ with leishmania and human 20S proteasomes.

The table represents RMSD values for alignments of Cα of individual chains *Tv*20-CP17 with *Leishmania tarentolae* 20S proteasome and human proteasome:

	RMSD *Leismania*7ZYJ	Chain ID	RMSD **Human 7PG9**		RMSD *Leismania* 7ZYJ	Chain ID	RMSD **Human 7PG9**
α1	1.252	A	**1.153**	β1	0.975	H	**0.976**
α2	1.219	B	**1.031**	β2	0.807	I	**0.989**
α3	0.985	C	**1.260**	β3	0.886	J	**0.898**
α4	1.048	D	**1.028**	β4	0.960	K	**1.289**
α5	1.252	E	**1.099**	β5	0.870	L	**1.010**
α6	1.116	F	**1.144**	β6	1.228	M	**1.050**
α7	1.121	G	**1.205**	β7	1.178	N	**1.489**

Overall RMSD values:
20S proteasome *Tv*20-CP17 with *Leishmania tarentolae* 20S proteasome **RMSD = 2.729**
20S proteasome *Tv*20-CP17 with human 20S proteasome **RMSD = 2.541**
The average RMSD values:
for all chains of *Leishmania tarentolae* 20S proteasome RMSD = 1.064
20S proteasome *Tv*20-CP17 with human 20S proteasome RMSD = 1.116*
(RMSD values were calculated for *Tv*20-CP17 (8P0T as a fix model) and human crystal structure (7PG9) for Cα atoms in the Pymol [The PyMOL Molecular Graphics System, Version 2.5.5 Schrödinger, LLC] using the command:align)

Supplementary Table 2. Cryo-EM statistics for data collection, refinement, and validation.

Deposited Cryo-EM structure	**Tv20S-MZB PDBID: 8OIX**	**Tv20S-CP-17 PDBID: 8P0T**
Microscope	Titan Krios	Titan Krios
Detector	Falcon 4i	Falcon 4i
Magnification (nominal)	165,000x	165,000x
Voltage (kV)	300	300
Spherical aberration	2.7 mm	2.7 mm
Total electron dose (e^-)	40	40
Defocus range (μm)	(-2.4)–(-0.9)	(-2.4)–(-0.9)
Exposure (s)	2.25	2.25
Pixel size (Å)	0.75	0.75
Number of Micrographs	7,983	10,037
Final number of particles	14,257	80,145
Map resolution (Å) [FSC threshold]	2.86 [$FSC_{0.143}$]	2.60 [$FSC_{0.143}$]
**Refinement**		
Initial model used (PDBID)	7ZYJ	8OIX
Symmetry during reconstruction	C2	C2
Validation		
MolProbity score	1.62	1.67
Clashscore, all-atom	11.17	11.90
Rotamer outliers	0.93 %	1.21 %
Ramachandran plot		
Favoured	97.73 %	97.92 %
Outliers	0.00 %	0.00 %
RMSD bonds		
Lenght (Å)	0.004	0.004
Angles (°)	0.611	0.659
**Model vs. Data**		
Ligands (no.)	4 (MZB)	4 (CP-17)
CC (mask/box/ligand)	0.87 / 0.68 / 0.80	0.84 / 0.64 / 0.77
B-factor protein (min/max/mean)	3.38 / 99.85 / 40.13	0.00 / 78.57 / 29.79
B-factor ligand (min/max/mean)	42.42 / 57.82 / 49.23	12.71 / 39.10 / 24.94
Resolution Estimates (Å)		
FSC model unmasked (0/0.143/0.5)	2.7 / 2.7 / 3.1	2.5 / 2.5 / 2.8

Refinment and model vs data values were calculated using Phenix 1.20.1-4487

Supplementary references

1 Robert, X. & Gouet, P. Deciphering key features in protein structures with the new ENDscript server. *Nucleic Acids Res* **42**, W320-324 (2014). https://doi.org:10.1093/nar/gku316

2 Meng, E. C. *et al.* UCSF ChimeraX: Tools for structure building and analysis. *Protein science : a publication of the Protein Society* **32**, e4792 (2023). https://doi.org:10.1002/pro.4792

3 Punjani, A., Rubinstein, J. L., Fleet, D. J. & Brubaker, M. A. cryoSPARC: algorithms for rapid unsupervised cryo-EM structure determination. *Nature methods* **14**, 290-296 (2017). https://doi.org:10.1038/nmeth.4169

Uncropped gels for Supplementary Figure 3a, b

denaturing gel - silver stained

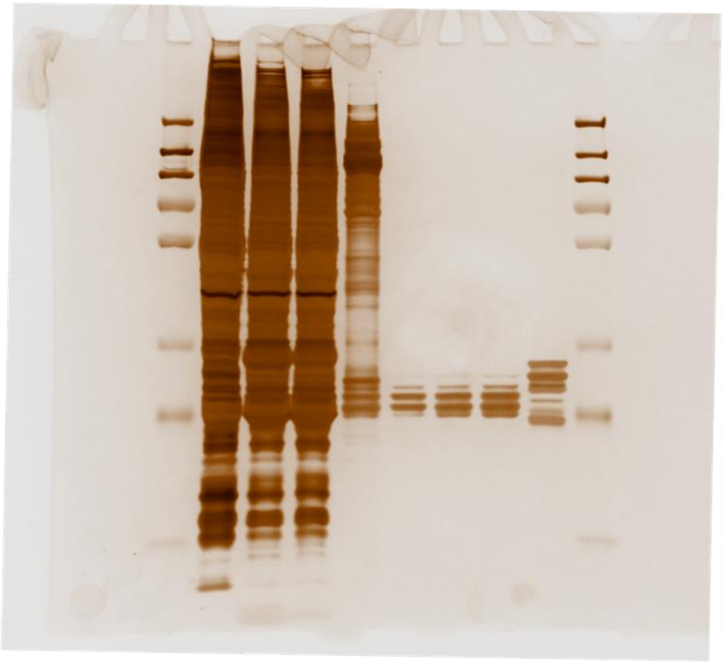

fluorescence

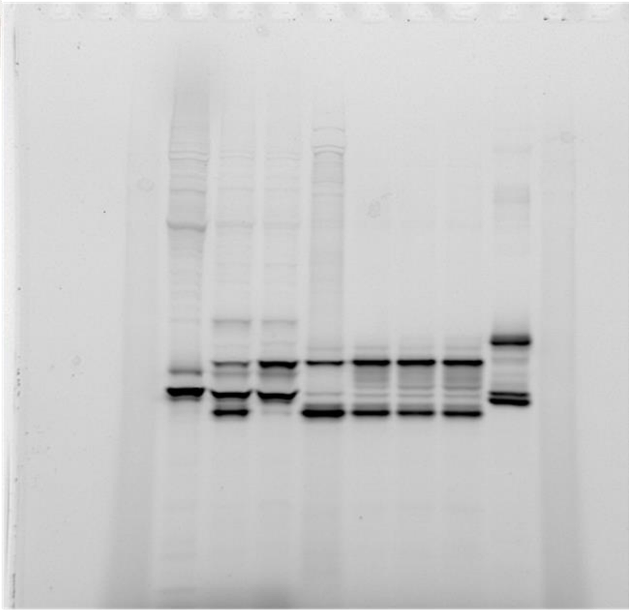

1. Sf lysate non-infected cells
2. alpha, beta, ump-1 coexpression
3. alpha, beta coexpression
4. 6. native Tv20S

5.-7. rTv20S

8. c20S

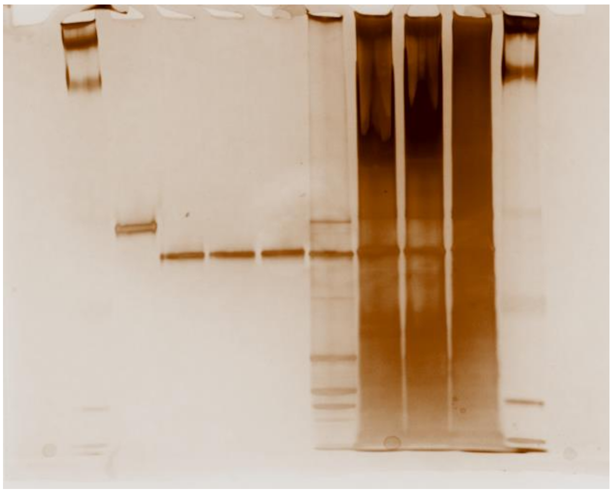

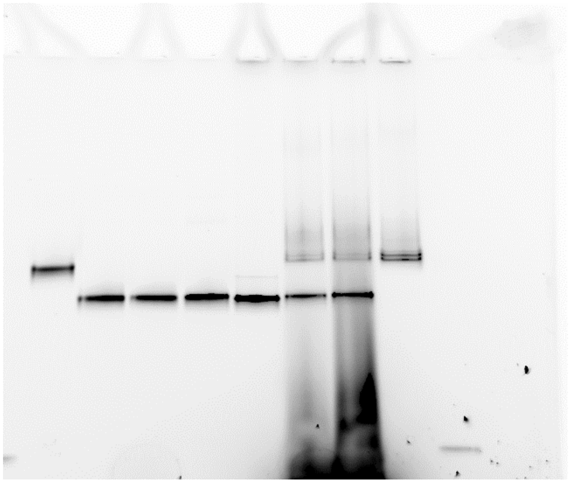

1. Sf lysate non-infected cells
2. alpha, beta, ump-1 coexpression
3. alpha, beta coexpression
4. 6. native Tv20S

5.-7. rTv20S

8. c20S

Uncropped gel for Supplementary Figure 3c

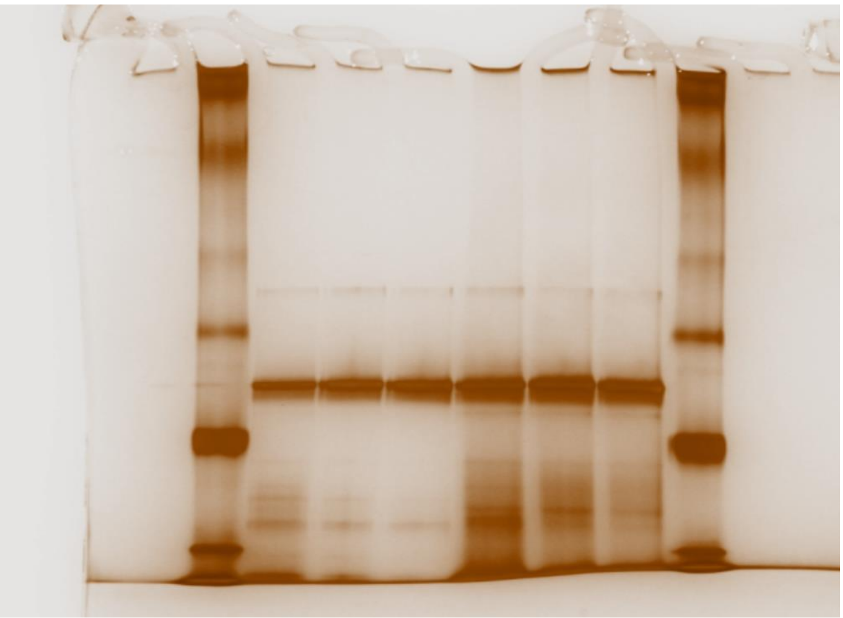

1) rTv20S + Ump time 0
2) rTv20S + Ump time 24 h
3) rTv20S + Ump time 72 h
4) rTv20S-Ump time 0
5) rTv20S - Ump time 24 h
6) rTv20S - Ump time 72 h

Uncropped gels for Supplementary Figure 3e

Native silver stain

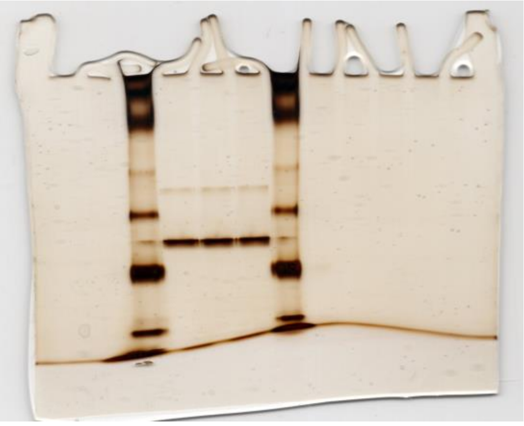

Fluorescence

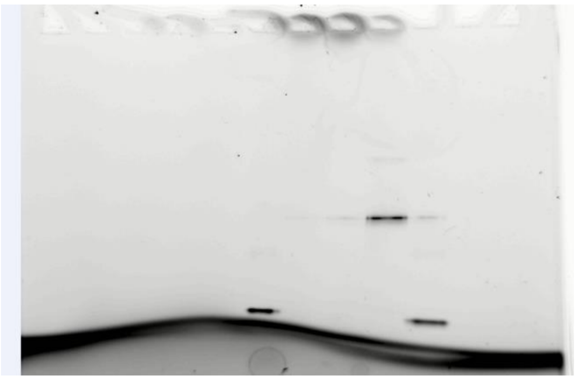

1) rTv20S+DMSO
2) rTv20S+CP17
3) rTv20S+MZB
