# Supplementary material for: Structural elucidation of recombinant Trichomonas vaginalis 20S proteasome bound to covalent inhibitors
Source: Nat Commun. 2024 Oct 4;15:8621. doi: 10.1038/s41467-024-53022-w (PMC11452676; doi:10.1038/s41467-024-53022-w)
Supplement: Supplementary file 3 — Description of Additional Supplementary Files [file 41467_2024_53022_MOESM3_ESM.pdf]

Description of Additional Supplementary Files

File Name: Supplementary Data 1
Description: This file contains proteomic analysis of rTv20S showing that the most abundant proteins correspond to each of the 14 subunits.
